# Centrioles generate two scaffolds with distinct biophysical properties to build mitotic centrosomes

**DOI:** 10.1126/sciadv.adq9549

**Published:** 2025-02-07

**Authors:** Siu-Shing Wong, Joao M. Monteiro, Chia-Chun Chang, Min Peng, Nada Mohamad, Thomas L. Steinacker, Bocheng Xiao, Saroj Saurya, Alan Wainman, Jordan W. Raff

**Affiliations:** ^1^ Sir William Dunn School of Pathology, University of Oxford, Oxford OX1 3RE, United Kingdom.; ^2^ The Max Planck Institute of Molecular Cell Biology and Genetics, Dresden, Germany.; ^3^ Institute of Biotechnology, National Taiwan University, 106 Taipei, Taiwan.

## Abstract

Mitotic centrosomes assemble when centrioles recruit large amounts of pericentriolar material (PCM) around themselves. The PCM comprises hundreds of proteins, and there is much debate about its physical nature. Here, we show that *Drosophila* Spd-2 (human CEP192) fluxes out from centrioles, recruiting Polo and Aurora A kinases to catalyze the assembly of two distinct mitotic-PCM scaffolds: a Polo-dependent Cnn scaffold, and an Aurora A–dependent TACC scaffold, which exhibit solid- and liquid-like behaviors, respectively. Both scaffolds can independently recruit PCM proteins, but both are required for proper centrosome assembly, with the Cnn scaffold providing mechanical strength, and the TACC scaffold concentrating centriole and centrosome proteins. Recruiting Spd-2 to synthetic beads injected into early embryos reconstitutes key aspects of mitotic centrosome assembly on the bead surface, and this depends on Spd-2’s ability to recruit Polo and Aurora A. Thus, Spd-2 orchestrates the assembly of two scaffolds, with distinct biophysical properties, that cooperate to build mitotic centrosomes in flies.

## Teaser

Spd-2 organizes the assembly of two distinct centriole-scaffolds to build mitotic centrosomes in *Drosophila*.

## INTRODUCTION

Centrosomes are membraneless organelles that form when centrioles recruit pericentriolar material (PCM) around themselves (*1*–*4*). The PCM probably comprises several hundred proteins (*5*, *6*), and centrosomes function as important microtubule (MT) organizing centers and cellular coordination hubs whose dysfunction has been linked to a plethora of human pathologies (*7*–*11*). Despite its molecular complexity, the mitotic PCM can assemble and disassemble rapidly as cells prepare to enter and exit mitosis, respectively (*12*–*15*), prompting much debate about the mitotic PCM’s biophysical nature (*16*–*20*). The mitotic PCM must be physically strong enough to not only resist the forces exerted by the spindle and astral MTs that it organizes but also provide an environment in which hundreds of proteins are concentrated and can potentially interact. In particular, there has been much debate about whether liquid-liquid phase separation (LLPS) may be important for mitotic centrosome assembly, as has been suggested for several other membraneless organelles (*21*, *22*).

In flies and worms, a relatively simple pathway of mitotic PCM assembly has been proposed. The centriole and PCM protein Spd-2/SPD-2 (fly/worm nomenclature) (*23*–*26*) recruits Polo/PLK-1 (*27*–*29*), which then phosphorylates Cnn/SPD-5 to stimulate the assembly of a macromolecular “scaffold” that can then recruit many different PCM “client” proteins (*27*, *30*–*34*). Cnn and SPD-5 have almost no sequence homology, but they are both large coiled-coil–rich proteins that can assemble into macromolecular scaffolding structures (*35*–*37*). This pathway appears to be widely conserved, and homologs of Spd-2/SPD-2 (*38*–*41*), Polo/PLK-1 (*42*–*46*), and Cnn (CEP192, PLK1 and CDK5RAP2 in humans, respectively) (*47*–*51*) have been implicated in mitotic centrosome assembly in many species.

Intriguingly, the fly Cnn scaffold appears solid-like (*36*), but purified recombinant worm SPD-5 forms condensates that exhibit transient liquid-like properties in vitro, leading to the suggestion that the centrosome in worms is a condensate that forms through LLPS and concentrates MT organizing proteins (*35*). Moreover, in mouse oocyte spindles—which lack canonical centrioles and centrosomes—a different protein, transforming acidic coiled-coil-containing protein 3 (TACC3), scaffolds a liquid-like spindle domain (LISD) that recruits many spindle components and is essential for meiotic spindle assembly; the LISD is also proposed to form via LLPS (*52*). The LISD is thought to be a specialized feature of centrosome-less meiotic spindles, as no LISD could be detected on mitotic spindles that were artificially induced to lack centrosomes (*52*). TACC proteins, however, are prominent components of mitotic centrosomes in many species (*53*, *54*), so we wondered whether centrosomes might organize a TACC-dependent LISD-like scaffold at normal mitotic spindles that form in the presence of centrosomes.

Here, we show that this is the case in fly embryos and that Spd-2 recruits not only Polo to stimulate the assembly of a solid-like Cnn scaffold but also Aurora A (AurA) to stimulate the assembly of a more liquid-like TACC scaffold that appears to be analogous to the mouse oocyte LISD. The Polo/Cnn and AurA/TACC scaffolds recruit PCM client proteins independently, but both are required for efficient mitotic centrosome assembly in embryos—with the Cnn scaffold providing mechanical strength and the TACC scaffold forming an extended network around the centrioles that concentrates key centriole and centrosome proteins. We show that centrioles generate an outward flux of Spd-2 molecules, and that recruiting Spd-2, but not Cnn or TACC, to the surface of synthetic beads injected into early embryos is sufficient to reconstitute several aspects of mitotic PCM assembly on the bead surface. Together, these studies demonstrate that Spd-2 acts as a nexus for centriole-driven mitotic centrosome assembly in fly embryos—fluxing outward from the mother centriole to recruit Polo and AurA to stimulate the assembly of two independent scaffolds that together drive the assembly and outward expansion of the mitotic PCM. Although the TACC scaffold is more liquid-like than the Cnn scaffold, our data do not distinguish whether the TACC scaffold is formed via LLPS.

## RESULTS

### *Drosophila* TACC and Cnn form independent centrosomal scaffolds that have different biophyiscal properties

**Fig. 1.**
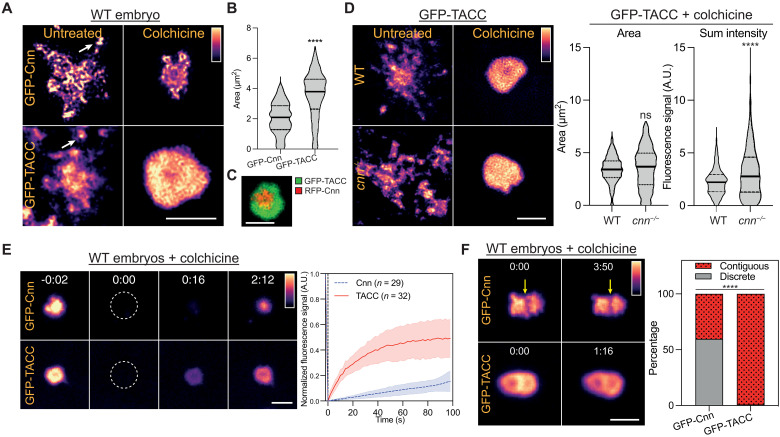
GFP-Cnn and GFP-TACC behave differently at centrosomes. (**A**) Images show the centrosomal distribution of GFP-Cnn or GFP-TACC in either untreated or colchicine-injected WT embryos. Arrows highlight “flares” breaking away from the main centrosome scaffold. (**B**) Violin plot shows the area (median ± quartiles) of the GFP-Cnn and GFP-TACC scaffolds in colchine-injected WT embryos. *N* = 13 to 20 embryos; *n* = ~400 to 800 centrosomes. (**C**) Image shows the distribution of RFP-Cnn and GFP-TACC at a typical centrosome in a colchicine-injected WT embryo. (**D**) Images show the distribution of GFP-TACC in either untreated or colchicine-injected WT or *cnn^−/−^* embryos. Violin plots show the area or fluorescence intensity (median ± quartiles) of the centrosomal GFP-TACC in these embryos *N* = 10 to 16 embryos; *n* = ~700 to 1300 centrosomes for each genotype. Statistical comparisons used Mann-Whitney test (****P < 0.0001; ns, not significant). (**E**) Images show the behavior of GFP-Cnn or GFP-TACC in a FRAP experiment in WT colchicine-injected embryos. Time (min:s) is indicated; centrosomes were bleached at *t* = 0:00. Graph shows each protein’s normalized fluorescence intensity recovery profile (means ± SD). *N* = 6 to 9 embryos; *n* = ~20 to 40 centrosomes. (**F**) Images show, and bar charts quantify, the distribution of GFP-Cnn or GFP-TACC at two closely abutted centrosomes that failed to separate properly in colchicine-injected WT embryos. GFP-Cnn usually occupies discrete centrosomal domains separated by a clear demarcation (arrow), while GFP-TACC usually forms a single continuous domain surrounding both centrosomes. *N* = 12 to 20 embryos; *n* = 100 to 300 centrosomes. Contingency significance (i.e., the significance of the difference in the proportion between the two groups) was calculated using a Fisher’s exact test (****P < 0.0001). A.U., arbitrary units. Scale bars, 2 μm.

To test whether *Drosophila* TACC might form an LISD-like scaffold at centrosomes, we first compared the centrosomal behavior of green fluorescent protein (GFP)–TACC and GFP-Cnn in early embryos, which cycle rapidly between S phase and mitosis with no Gap phases. In these embryos, the centrosomes essentially continuously recruit a mitotic-like PCM around the centrioles (*55*, *56*). As reported previously, both GFP-TACC and GFP-Cnn spread out from the centrosome along microtubules, often breaking off from the centrosome periphery as “flares” (*57*, *58*) (arrows, Fig. 1A, left panels). When microtubules were depolymerized using colchicine, flaring was suppressed, and GFP-Cnn condensed into a heterogeneous scaffold with irregular edges, whereas GFP-TACC organized a more uniform structure with smoother edges (Fig. 1A, right panels). The TACC structure extended beyond the Cnn scaffold (Fig. 1B)—seen most clearly in embryos coexpressing GFP-TACC and red fluorescent protein (RFP)–Cnn (Fig. 1C). This suggests that the TACC structure has some mechanical integrity that is independent of the Cnn scaffold, and we hereafter refer to it as a TACC scaffold.

To test whether the TACC scaffold assembled upon the Cnn scaffold, we expressed GFP-TACC in embryos from c*nn*^−/−^ mutant females (hereafter *cnn^−/−^* embryos). GFP-TACC was still detected at centrosomes in *cnn^−/−^* embryos but formed more extensive flares that spread out over a broader area (Fig. 1D, left panels, and movie S1). When flaring was suppressed by colchicine, however, GFP-TACC recruitment to centrosomes was, if anything, slightly enhanced in *cnn^−/−^* embryos (Fig. 1D). A fluorescence recovery after photobleaching (FRAP) analysis revealed that GFP-TACC was recruited to centrosomes with similar kinetics in WT and *cnn^−/−^* embryos (fig. S1). Thus, the TACC scaffold can form independently of the Cnn scaffold, but the latter appears to normally stabilize the former to prevent its dispersion on centrosomal microtubules.

**Fig. 2.**
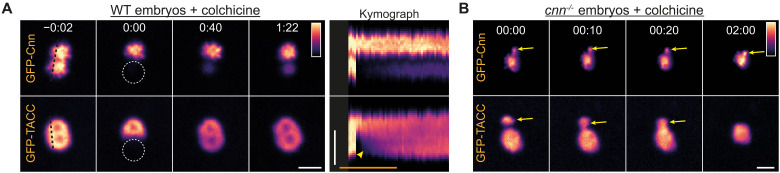
GFP-Cnn and GFP-TACC exhibit solid-like and liquid-like behavior, respectively. (**A**) Images show the FRAP behaviour of GFP-Cnn or GFP-TACC at closely paired centrosomes in which one centrosome has been bleached (at *t* = 0:00). The kymograph shows how fluorescence intensity changes over time along the black dotted lines indicated in the prebleach images (*t* = −0:02). The arrow highlights the gradient of fluorescence intensity observed as fluorescent GFP-TACC molecules from the unbleached centrosome move toward the bleached centrosome. (**B**) Images show how flares (arrows) of GFP-Cnn do not fuse with the main centrosome scaffold, but these fusion events are readily detected with flares of GFP-TACC. Scale bars, 2 μm.

In colchicine-injected wild-type (WT) embryos, FRAP analysis showed that centrosomal GFP-Cnn fluorescence recovered slowly and primarily around the central region of the centrosome, while GFP-TACC fluorescence recovered more quickly and evenly throughout the centrosomal region, indicating a constant exchange between the centrosomal and cytoplasmic fractions (Fig. 1E and movie S2). In these colchicine-injected embryos, the duplicated centrosomes often only partially separated. In these instances, GFP-Cnn usually formed discrete scaffolds around each centrosome with a stable boundary between them (Fig. 1F, arrow), while GFP-TACC typically formed a continuous scaffold with no clear demarcation between the two centrosomes (Fig. 1F and movie S3). To examine the ability of the GFP-Cnn and GFP-TACC molecules to internally rearrange within these structures, we photobleached one of the centrosomes within such a pair and observed the pattern of fluorescence recovery (Fig. 2A). Kymographs of these partially photobleached pairs revealed that GFP-Cnn molecules were largely immobile, and we could detect no movement of fluorescent molecules from the unbleached centrosome toward the bleached centrosome (Fig. 2A and movie S4). In contrast, we readily detected the movement of fluorescent GFP-TACC molecules from the unbleached centrosome toward the bleached centrosome—observed as a gradient on the kymograph (Fig. 2A, arrow and movie S4). The gradual reduction in fluorescence intensity of the unbleached centrosome also indicated that bleached GFP-TACC molecules were moving toward the unbleached centrosome, which we did not observe with GFP-Cnn molecules (fig. S2). Last, in *cnn^−/−^* embryos injected with colchicine, GFP-TACC flares often fused with the centrosomal GFP-TACC scaffold, while similarly positioned GFP-Cnn flares did not (Fig. 2B). Together, these data indicate that the centrosomal Cnn scaffold exhibits a more solid-like behavior, whereas the TACC scaffold exhibits a more liquid-like behavior.

### The centrosomal TACC scaffold is related to the mammalian LISD

**Fig. 3.**
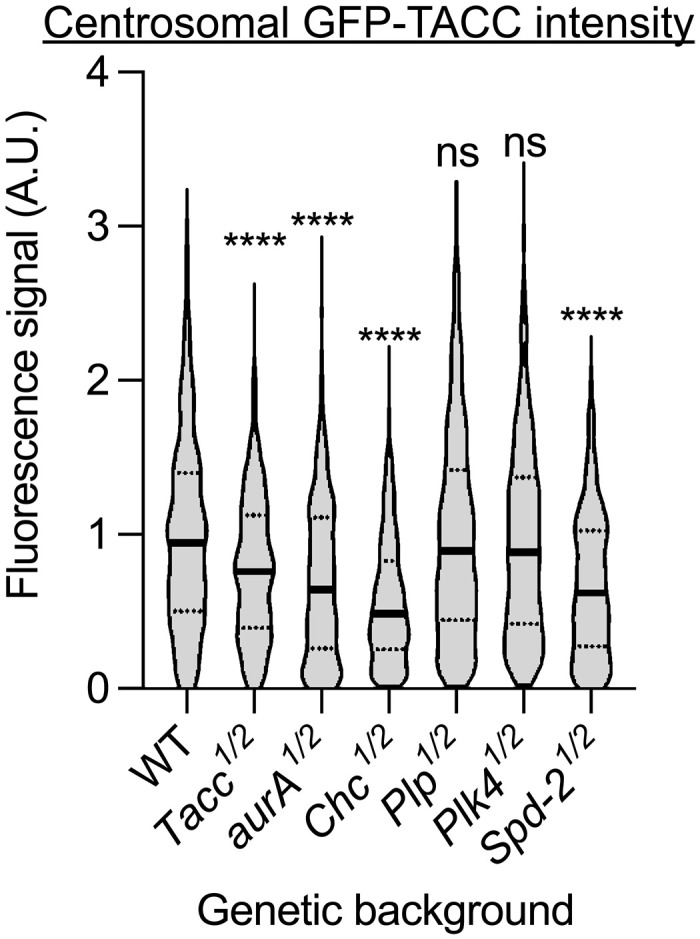
Analysis of the molecular requirements for TACC-scaffold assembly. Violin plot quantifies the centrosomal fluorescence intensity (median ± quartiles) of centrosomal GFP-TACC in living embryos laid by mothers that are heterozygous for null mutations in the indicated genes (so the embryos contain approximately half the normal amount of each protein—see Materials and Methods). *N* = 10 to 15 embryos; *n* = 500 to 1000 centrosomes. Statistical significance was calculated using a Kruskal-Wallis test, followed by a Dunn’s multiple comparison (****P < 0.0001). ns, not significant.

TACC3, AURA, and Clathrin heavy chain 17 (CHC17) are all critical for LISD assembly in the mouse female meiotic spindle (*52*, *59*, *60*). To test their requirement for fly centrosomal TACC scaffold assembly, we quantified centrosomal GFP-TACC levels in embryos from mothers with a halved genetic dosage of *Tacc*, *aurA*, or *Chc*. These experiments were conducted in *cnn^−/−^* embryos injected with colchicine to avoid any confounding effects from the Cnn scaffold. These perturbations significantly reduced the size of the TACC scaffold, while similarly halving the genetic dose of the Pericentrin-like protein (Plp) or Plk4—proteins involved in centrosome and centriole assembly (*61*–*64*) that are not components of the LISD (*52*)—did not (Fig. 3). Thus, the fly centrosomal TACC scaffold and the mouse LISD appear to be related.

**Fig. 4.**
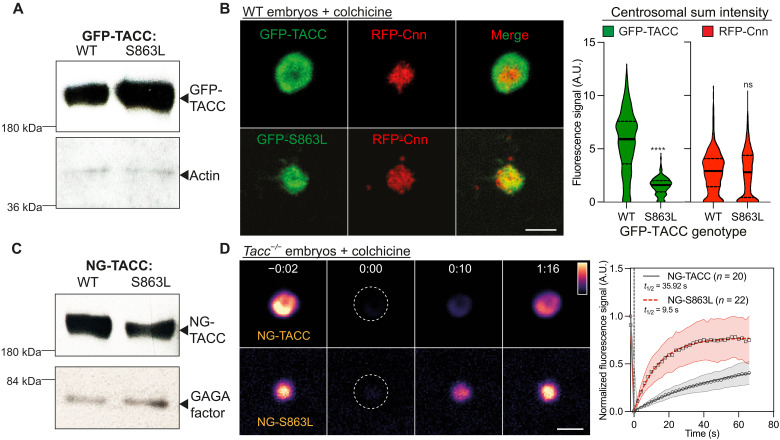
TACC phosphorylation on Ser^863^ promotes scaffold assembly. (**A**) 0- to 2-hour-old embryos expressing either WT GFP-TACC or GFP-TACC-S863L (*31*) were blotted with antibodies against GFP. Actin is shown as a loading control. (**B**) Images show the centrosomal localisation of WT GFP-TACC or the GFP-TACC-S863L mutant in WT embryos expressing RFP-Cnn. Violin plots quantify the total centrosomal fluorescence intensities (median ± quartiles) of GFP-TACC and GFP-S863L (left) or RFP-Cnn (right) in these embryos. *N* = 10 embryos; *n* = 800 to 900 centrosomes for each group. Statistical comparisons used a Mann-Whitney test (****P < 0.0001). ns, not significant. (**C**) 0- to 2-hour-old embryos expressing either WT NG-TACC or NG-TACC-S863L from the ubiquitin (Ubq) promoter were blotted with antibodies against TACC. GAGA factor is shown as a loading control. (**D**) Images show the behaviors of NG-TACC or NG-S863L in a FRAP experiment in colchicine-injected *Tacc^−/−^* embryos. Time (min:s) is indicated; centrosomes were bleached at *t* = 0:00. Graph shows each protein’s normalized fluorescence intensity recovery profile (means ± SD). Fitted lines and their parameters were generated using a one-phase association model (Graphpad Prism). *N* = 4 to 9 embryos; *n* = 20 to 30 centrosomes for each group. Scale bars, 2 μm.

AurA/AURKA phosphorylates TACC/TACC3 on a conserved serine, S863/S558 in flies/humans (*65*, *66*), allowing TACC3 to interact with CHC (*67*–*69*). This phosphorylation appears to be important for TACC scaffold assembly, as we found that a nonphosphorylatable GFP-TACC-S863L mutant (*65*) was still recruited to centrosomes, but it appeared unable to form a TACC scaffold as the mutant protein no longer formed a structure that extended outward beyond the Cnn scaffold (Fig. 4, A and B). Moreover, a FRAP analysis of mNeonGreen (NG)—TACC-WT and NG-TACC-S863L in *Tacc^−/−^* embryos revealed that TACC-S863L exhibited faster turnover than TACC-WT, suggesting that although TACC-S863L can still be recruited to centrosomes, it cannot efficiently form a TACC scaffold (Fig. 4, C and D). Thus, AurA phosphorylates Ser^863^ of TACC to promote TACC scaffold assembly, likely by promoting TACC’s interaction with CHC. The Cnn scaffold appeared largely unperturbed in embryos expressing GFP-TACC-S863L, suggesting that it can assemble largely independently of the TACC scaffold (Fig. 4B).

### Spd-2 helps recruit AurA and Polo to centrosomes

**Fig. 5.**
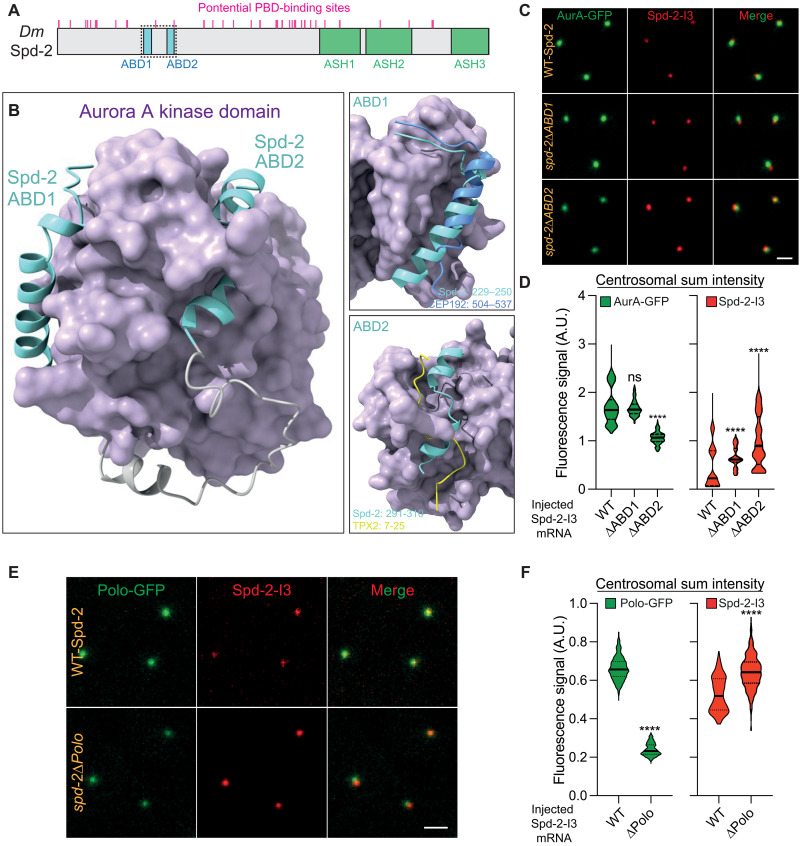
Testing potential interactions between Spd-2 and AurA or Polo. (**A**) Schematic representation of *Drosophila* Spd-2 highlighting the position of the two potential AurA binding domains (ABD1 and ABD2) (cyan boxes), the three C-terminal ASPM-SPD-2-Hydin (ASH) domains (green boxes), and the multiple potential Polo-Box binding motifs examined previously (magenta lines) (*6*). The fragment of Spd-2 shown in the predicted structure below is indicated by a black dotted box. (**B**) ColabFold predicted structure of Spd-2_229-310_ (ABD1 and ABD2 in cyan with gray linker region) bound to the AurA kinase domain (indigo surface). The two putative interaction sites (ABD1_229–250_ and ABD2_291–310_) are shown in the boxes on the right overlayed with the previously described crystal structure of either human CEP192_504–537_ (blue, top box) or *Xenopus* TPX-2_7-25_ (yellow, bottom box) bound to AURKA (overlayed here on the *Drosophila* AurA structure). See fig. S3 for a more detailed analysis. (**C** and **D**) Images show, and violin plots quantify (median ± quartile), centrosome fluorescence intensity in embryos expressing AurA-GFP and injected with mRNA encoding mScarlet-I3-fusions to either WT-Spd-2 or mutant forms of Spd-2 in which the putative ABD’s have been deleted (Spd-2∆ABD1 and Spd-2∆ABD2). *N* = 9 embryos; *n* = 400 to 500 centrosomes for each group. Statistical significance was calculated using a Kruskal-Wallis test, followed by a Dunn’s multiple comparison (****P < 0.0001). ns, not significant. (**E** and **F**) Images show, and violin plots quantify (median ± quartile), centrosome fluorescence intensity in embryos expressing Polo-GFP and injected with mRNA encoding mScarlet-I3-fusions to either WT-Spd-2 or a mutant form of Spd-2 in which all 34 of the potential Polo-Box-domain (PBD) binding motifs (S-S/T) were mutated to T-S/T, which prevents PBD-binding (*6*). The positions of the 34 S-S/T motifs present in Spd-2 is indicated in (A) (magenta lines). *N* = 10 to 11 embryos; *n* = 500 to 600 centrosomes for each group. Statistical significance was calculated using a Mann-Whitney (****P < 0.0001). Scale bars, 2 μm.

As AurA phosphorylation is critical for TACC scaffold assembly, we wondered whether Spd-2 might also play a part in TACC scaffold assembly, as Spd-2/CEP192 proteins recruit AURKA, as well as PLK1, to centrosomes in vertebrates (*45*, *46*). Supporting this possibility, halving the genetic dose of *Spd-2* reduced TACC scaffold assembly similarly to halving the dose of *aurA* (Fig. 3). In vertebrates, the CEP192/AURKA interaction is well characterized (*40*, *45*, *46*) with a recently described crystal structure of an interaction interface (*70*). It is unclear, however, if the fly/worm Spd-2/SPD-2 proteins interact with AurA (*71*). Using AlphaFold2-Multimer, we identified a high-confidence interaction (iPTM = 0.59) between *Drosophila* Spd-2_229–310_ and AurA’s kinase domain (AurA_155–421_) (Fig. 5, A and B). This involves two independent interfaces—Spd-2_229–250_ and Spd-2_291–310_, which we term AurA-binding domain (ABD) 1 and 2, respectively—wrapping around the surface of the kinase domain. In this predicted structure, Spd-2-ABD1 and Spd-2-ABD2 bind to similar regions on AurA/AURKA as human CEP192 (*70*) and human TPX2 (*72*), respectively, although there is only limited sequence homology between these binding domains (fig. S3). A very recent study also identified a second AURKA interaction interface similar to ABD2 in human CEP192, suggesting that the bipartite interaction interface between Spd-2/CEP192 and AurA/AURKA is conserved in flies and humans (*73*).

To test whether ABD1 or ABD2 help recruit AurA to centrosomes in flies, we injected mRNA encoding mScarlet-I3-fusions (I3) (*74*) to either WT Spd-2 or forms of Spd-2 with ABD1 or ABD2 deletions into embryos expressing AurA-GFP. In this assay, the injected mRNA is quickly translated, and the expressed I3-fusions compete with the endogenous unlabeled protein to bind to the centrosomes (*75*). AurA-GFP recruitment was unchanged or significantly reduced in the presence of Spd-2∆ABD1 or Spd-2∆ABD2, respectively (Fig. 5, C and D), indicating that ABD2 plays an important part in recruiting AurA to centrosomes. The data we present below, however, suggest that ABD1 probably also has a role.

We then also used this assay to confirm the central role of Spd-2 in recruiting Polo to centrosomes. We previously showed that Spd-2 recruits Polo to centrosomes via Polo’s Polo-Box domain (PBD) by mutating all the potential PBD-binding S-S/T motifs in Spd-2 (indicated by magenta lines in Fig. 5A) to T-S/T (generating Spd-2∆Polo, previously termed Spd-2-ALL, which does not recruit Polo) (*28*). A Spd-2∆Polo-I3 fusion strongly reduced Polo recruitment compared to WT Spd-2-I3 in the mRNA injection assay (Fig. 5, E and F). Thus, as in vertebrates, *Drosophila* Spd-2 helps recruit both Polo and AurA to centrosomes. Intriguingly, we note that the centrosomal recruitment of mutant forms of Spd-2 that cannot recruit AurA or Polo efficiently was actually increased (Fig. 5, D and F, red graphs). We do not know why this is the case, but we speculate that the ability of Spd-2 molecules to recruit these kinases may allow Spd-2 molecules to more efficiently leave the centrosome (see below).

### Spd-2 molecules incorporate into the PCM close to the centrioles and then flux outward

**Fig. 6.**
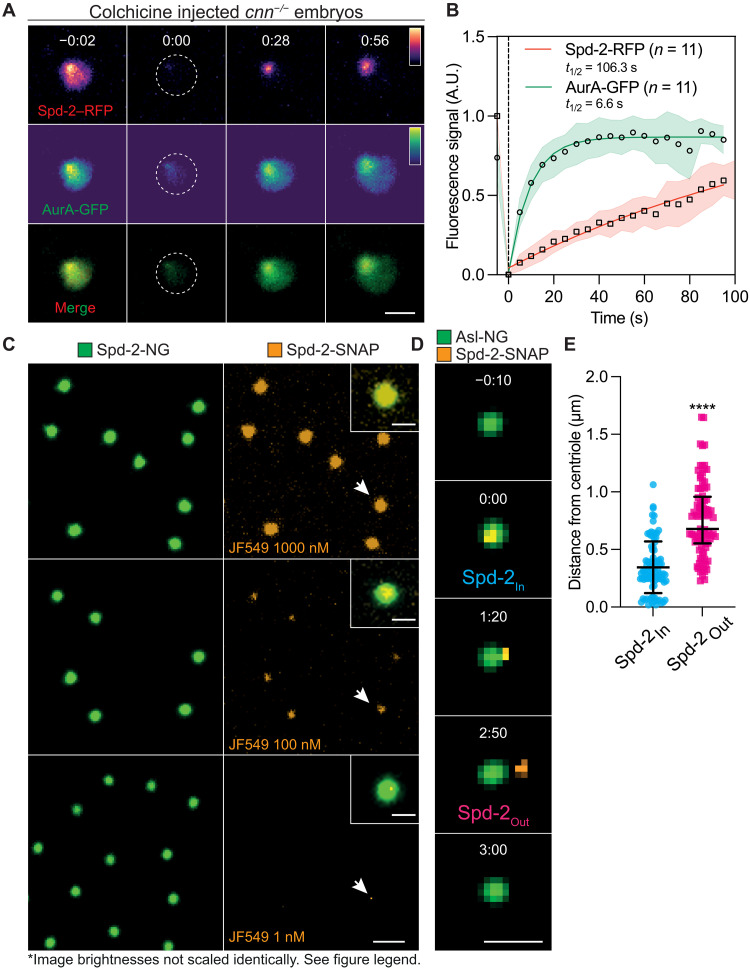
Spd-2 molecules flux outwards from the centrioles. (**A**) Images show the behavior of Spd-2-RFP and AurA-GFP in a FRAP experiment in colchicine-injected *cnn^−/−^* embryos. Time (min:s) is indicated; centrosomes were bleached at *t* = 0:00 . (**B**) Graph shows each protein’s normalized fluorescence intensity recovery profile (mean ± SD). *N* = 9 embryos; *n* = 11centrosomes. (**C**) Images show the centrosomes in embryos coexpressing Spd-2-NG and Spd-2-SNAP and injected with different concentrations of the JF-549 SNAP-ligand. The lower the concentration of injected ligand, the sparser the labeling of the centrosomes. The centrosomes highlighted with white arrows are shown in the enlarged insets. (**D**) Images show stills from a movie tracking a single molecule of fluorescently labeled Spd-2-SNAP binding to, and then unbinding from, a mother centriole (labeled with Asl-NG). Time (min:s) relative to the first detection of the single particle at the centriole (*t* = 0:00) is indicated. (**E**) Scatter plot shows the distance (median ± quartiles) between single molecules of fluorescently labeled Spd-2-SNAP and the center of the mother centriole at the time the molecules initially bind to the centrosome (*Spd-2_In_*) and at the time they leave the centrosome (*Spd-2_Out_*). *N* = 89 single molecules. Scale bars, (A and D) 2 μm and (C) 4 μm (inset = 2 μm).

In colchicine-injected *cnn^−/−^* embryos coexpressing Spd-2-RFP and AurA-GFP, both proteins exhibited a very similar localization, concentrating around the centrioles and spreading throughout the TACC scaffold region (Fig. 6A; *t* = −0:02). A FRAP analysis revealed, however, that they exhibited very different dynamics: Spd-2-RFP fluorescence initially recovered slowly at the centrioles and then appeared to spread outwards through the TACC scaffold, while AurA-GFP fluorescence recovered more rapidly not only around the centrioles but also throughout the TACC scaffold region (Fig. 6, A and B). This suggests that Spd-2 molecules can only be incorporated into the scaffold by the centrioles (from where they then flux outward), while AurA can be recruited into the scaffold by Spd-2 molecules that are concentrated around the centrioles and also by those spread throughout the scaffold region. In this interpretation, the centrioles serve as a unique source of Spd-2 and so as an essential driver of scaffold assembly. In support of this, we observed instances in colchicine-injected *cnn^−/−^* embryos where the GFP-TACC scaffolds became separated from the Spd-2-RFP–generating centrioles; this led to de novo TACC scaffold assembly around the centrioles and the eventual disassembly of TACC scaffolds that had lost their connection to the centrioles (fig. S4). Thus, de novo TACC scaffold assembly appears to require the centrioles, presumably because they act as a source of Spd-2, and so AurA.

In this scenario, the outward flux of Spd-2 from the centrioles might help to drive the expansion of the AurA/TACC scaffold around the centrioles, as we have postulated to be the case for the expansion of the Polo/Cnn scaffold (*4*, *34*). The outward flux of Spd-2, however, remains controversial and unproven. This pattern of Spd-2 fluorescence recovery (first around the centrioles and then “spreading” outward through the PCM—Fig. 6A) might reflect a gradient in the turnover rate of Spd-2–binding sites within the PCM rather than a physical outward flux of Spd-2 molecules. Moreover, no outward flux of SPD-2 was observed in *Caenorhabditis elegans* embryos (*31*).

To directly test the Spd-2–flux hypothesis in flies, we examined the behavior of single Spd-2 molecules in living embryos. We generated transgenic lines expressing Spd-2 fused to a soluble NSF attachment protein (SNAP)–tag (*76*), which can be covalently coupled to the fluorescent SNAP-ligand JF-549 (*77*). When injected at a concentration of 1 μM into *Drosophila* embryos expressing Spd-2-SNAP and Spd-2-NG, the SNAP-ligand appeared to label most Spd-2-SNAP molecules, but the labeled fraction decreased as we lowered the ligand concentration (Fig. 6C). At 1 nM, most of the centrosomes were not detectably labeled at all, while ~5 to 10% of the centrosomes contained what appeared to be a single fluorescent spot, presumably generated by a single fluorescent Spd-2-SNAP molecule (Fig. 6C, arrow, bottom panels).

We injected embryos expressing Spd-2-SNAP and Asl-NG (a mother centriole marker) (*78*) with 1 nM JF-549 and conducted time-lapse imaging to track the trajectory of individual Spd-2-SNAP molecule’s after they bound to centrosomes. We identified single-molecule binding events (see Materials and Methods) and computed the distance between the mother centriole and each Spd-2-SNAP molecule over time (Fig. 6, D and E). On average, these single Spd-2-SNAP molecules bound into the PCM (*Spd-2*_In_) close to the centriole (345 ± 225 nm, mean ± SD) and dissociated from the PCM (*Spd-2*_Out_) further away from the centriole (762 ± 322 nm) (Fig. 6, D and E, and movie S5). Thus, Spd-2 molecules flux outward from the centriole in fly embryos.

### The TACC scaffold concentrates centrosome proteins

**Fig. 7.**
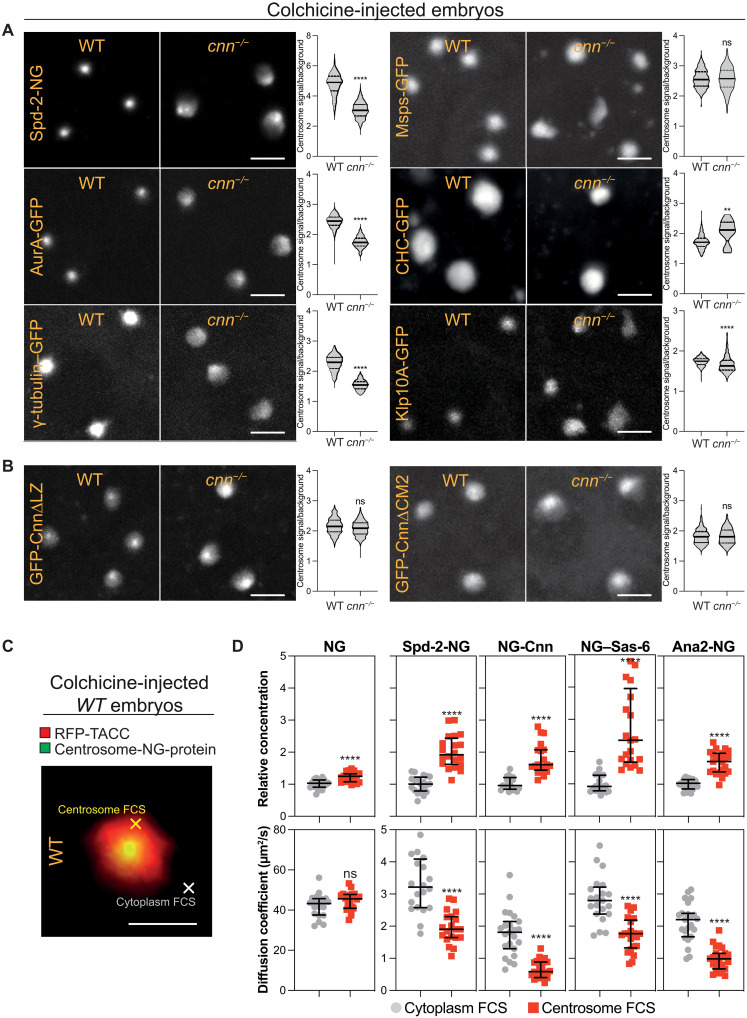
The TACC scaffold concentrates centriole and centrosome components. (**A**) Images show the accumulation of fluorescent-fusions to various proteins at centrosomes in WT or *cnn^−/−^* embryos treated with colchicine. Violin plots quantify (median ± quartiles) the fold enrichment of the proteins at the centrosome compared to the cytoplasm. *N* = 3 to 10 embryos; *n* = 60 to 1000 centrosomes for each group. (**B**) These panels show the same as in (A) but for GFP-fusions to Cnn deletion mutants (∆LZ or ∆CM2) that cannot form a Cnn scaffold. *N* = 10 to 11 embryos; *n* = 400 to 900 centrosomes for each group. (**C**) Image shows a centrosome in a WT embryo expressing RFP-TACC and Ana2-NG and injected with colchicine. This illustrates the typical areas that were analyzed by FCS in the outer regions of the centrosome (yellow cross) or the nearby cytoplasm (white cross). Scale bar, 2 μm. (**D**) Scatter plots show the FCS-measured concentration (top plots) or diffusion rate (bottom plots) (median ± quartiles) of NG or various NG-fusions in the cytoplasm (gray circles) or in the outer regions of the centrosome (red squares) in WT embryos injected with colchicine. *N* = 20 to 25 embryos. Statistical significance was calculated using a Mann-Whitney test (****P < 0.0001). ns, not significant.

To determine whether, like the LISD in mouse oocyte spindles (*52*), the TACC scaffold can recruit centrosome/spindle proteins, we compared the centrosomal enrichment of several fluorescently tagged centrosomal proteins in both WT and *cnn^−/−^* embryos injected with colchicine. All tested proteins were significantly enriched at centrosomes in both WT and *cnn^−/−^* embryos, indicating that the TACC scaffold can recruit proteins to centrosomes independently of the Cnn scaffold (Fig. 7A). Spd-2-NG, AurA-GFP, and γ-tubulin-GFP were more enriched at centrosomes in WT embryos than in *cnn^−/−^* embryos, indicating that the Cnn scaffold plays a direct role in recruiting these proteins to centrosomes. In contrast, the centrosomal levels of Msps-GFP and CHC-GFP were not perturbed or were even slightly enhanced in *cnn^−/−^* embryos, with Klp10A exhibiting only a relatively subtle perturbation. These observations suggest that the Cnn scaffold may have a direct role in recruiting only a subset of PCM-clients.

We also examined the recruitment of two mutant forms of Cnn that do not efficiently assemble the Cnn scaffold (GFP-Cnn∆LZ and GFP-Cnn∆CM2) (*36*). Both mutants were similarly enriched in the TACC scaffold in colchicine-injected WT and *cnn^−/−^* embryos (Fig. 7B). This is important, as it demonstrates that the TACC scaffold can concentrate Cnn molecules independently of the Cnn scaffold and independently of the ability of the Cnn molecules to form a Cnn scaffold. Thus, the TACC scaffold may locally concentrate Cnn molecules to promote the efficiency of Cnn scaffold assembly.

To probe the microenvironment created by the TACC scaffold, we used fluorescence correlation spectroscopy (FCS) to compare the concentration and diffusion rates of Spd-2 and Cnn in the cytoplasm (Fig. 7C, white cross) and within the TACC scaffold region that extended outward beyond the Cnn scaffold in colchicine-treated embryos (Fig. 7C, yellow cross). As the PCM can also promote centriole assembly (*79*), we also examined the behavior of the centriole building blocks NG-Sas-6 and Ana2-NG (*80*, *81*). As a control, we first analyzed unfused-NG molecules; these were slightly enriched in the TACC-scaffold region, but their diffusion rate within the scaffold was not significantly altered compared to the cytoplasm (Fig. 7D). In contrast, all the centriole and centrosome proteins were more enriched and had significantly reduced diffusion rates, within the TACC scaffold (Fig. 7D). Similar results were observed in *cnn^−/−^* embryos, demonstrating that this property of the TACC scaffold does not depend on the Cnn scaffold (fig. S5). We conclude that the TACC scaffold concentrates key centriole and centrosome proteins, at least in part, by slowing their diffusion within the scaffold. This is presumably because these proteins can bind and unbind to either the TACC scaffold itself or to other elements concentrated within the TACC scaffold.

### Reconstituting elements of mitotic PCM assembly on the surface of synthetic beads

**Fig. 8.**
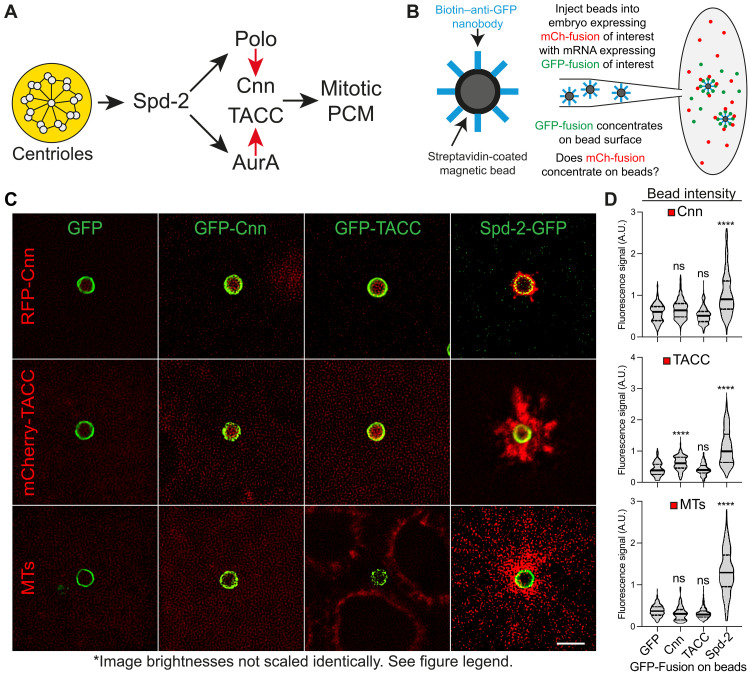
Reconstituting aspects of mitotic PCM assembly on the surface of synthetic beads. (**A**) Schematic illustrates the proposed pathway of mitotic-PCM-scaffold assembly. Centrioles provide a source of Spd-2 that fluxes outwards, recruiting AurA and Polo, which then phosphorylate TACC and Cnn (red arrows), respectively, to initiate the assembly of scaffolds that support the assembly of the mitotic PCM. (**B**) Schematic illustrates the synthetic bead injection assay. (**C** and **D**) Images show, and violin plots quantify (median ± quartiles), the red fluorescent signal—RFP-Cnn, mCherry-TACC (as markers of the PCM-scaffolds), or Jupiter-mCherry (as a marker of MTs)—on the beads when the beads are bound to GFP or the various GFP-fusions. Statistical significance was calculated by an ordinary one-way analysis of variance (ANOVA), followed by a Tukey’s multiple comparison (****P < 0.0001). ns, not significant. Note that, for unknown reasons, the GFP-Cnn bound beads appear to recruit significantly more mCherry-TACC than controls, but this recruitment is not nearly as strong as that observed with the Spd-2-GFP–coated beads. Scale bar, 2 μm.

As described in Introduction, there seems to be a relatively simple “core” pathway that drives mitotic centrosome assembly in fly embryos (Fig. 8A). In this schematic, the mother centriole acts as a platform that supports the Spd-2–dependent assembly of a Polo/Cnn scaffold, and our current data suggest also a Spd-2–dependent AurA/TACC scaffold. We wondered if we could reconstitute this pathway using synthetic beads as an alternative platform. To this end, we coupled streptavidin-coated magnetic beads to a biotinylated anti-GFP-Nanobody and injected these together with mRNA encoding GFP, GFP-Cnn, GFP-TACC, or Spd-2-GFP into embryos expressing either RFP-Cnn or mCherry-TACC (to monitor scaffold assembly) or Jupiter-mCherry (to monitor microtubule assembly) (Fig. 8B).

The synthetic beads recruited all the GFP or GFP-fusions to similar extents (Fig. 8C), but only the Spd-2-GFP beads also recruited RFP-Cnn, mCherry-TACC, and MTs (Fig. 8, C and D), and the dynamics of the MTs organized by the Spd-2–beads cycled in synchrony with the dynamics of the endogenous centrosomes (movie S6). Spd-2-GFP did not extend outward around the bead surface (presumably because it is bound to the high-affinity anti-GFP-nanobody and so cannot flux outwards), and the Cnn scaffold also remained closely associated with the bead surface; in contrast, the TACC scaffold spread outward around the bead to a much greater extent (Fig. 8C). Thus, the outward flux of Spd-2 at the centriole may be required for the outward expansion of the solid-like Cnn scaffold, but the more liquid-like TACC scaffold can expand outward independently of Spd-2 flux, at least to some extent. As at centrosomes (Fig. 1A), the TACC scaffold associated with the Spd-2 beads appeared to be pulled outward along the MTs (Fig. 8C), but it still formed an extensive and more rounded scaffold around the beads when the MTs were depolymerized with colchicine (fig. S6). Thus, the outward expansion of the TACC scaffold around centrosomes and beads is not primarily driven by MT-pulling forces.

### PCM assembly on Spd-2 beads requires Polo and AurA recuitment

**Fig. 9.**
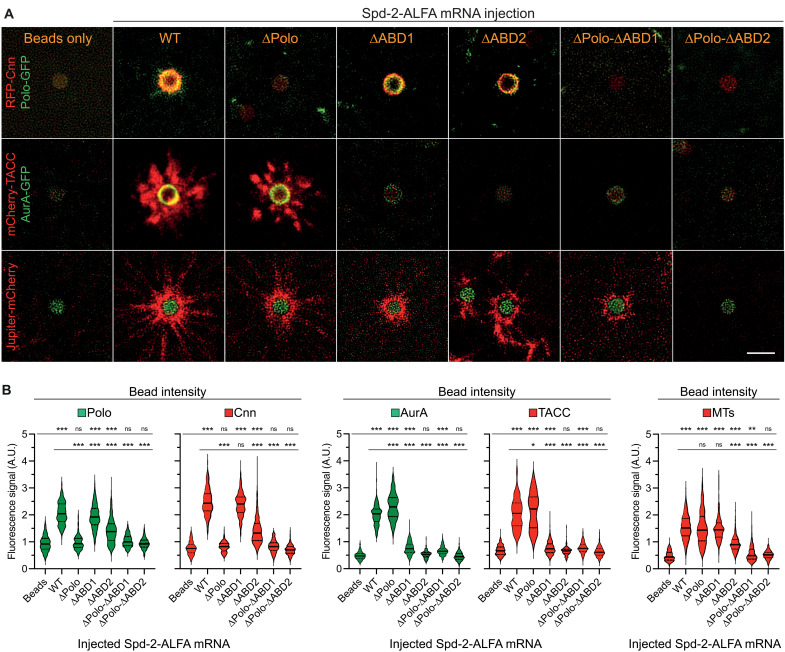
Spd-2 can recruit Polo/Cnn and AurA/TACC at least partially independently to organize PCM and MTs at synthetic beads. (**A**) Images show ALFA-tag–Nanobody–coupled synthetic beads that have been injected into embryos expressing either RFP-Cnn and Polo-GFP (top), mCherry-TACC and AurA-GFP (middle), or Jupiter-mCherry (bottom), together with mRNA encoding ALFA-tagged versions of either WT Spd-2 or Spd-2 mutants that are unable to efficiently recruit Polo (∆Polo) or AurA (∆ABD1 or ∆ABD2). Scale bar, 2 μm. (**B**) Violin plots quantify the fluorescent signal (median ± quartiles) of the various green or red fluorescent fusions (indicated at the top of each plot) that are recruited to the beads when coupled to the different ALFA-tagged Spd-2–fusions (indicated at the base of each plot). Statistical significance was calculated by an ordinary one-way ANOVA, followed by a Tukey’s multiple comparison (*P < 0.05; **P < 0.01; ***P < 0.001; ns, not significant).

To test whether the recruitment of Polo and/or AurA was necessary for the Spd-2–coated beads to organize Cnn/TACC scaffolds, we conjugated the streptavidin-coated beads to a biotinylated anti-ALFA-tag-Nanobody (the ALFA-tag is not fluorescent) (*82*) and co-injected mRNA encoding WT or various mutant ALFA-tagged versions of Spd-2 into embryos expressing either Polo-GFP and RFP-Cnn, or AurA-GFP and mCherry-TACC, or the MT-marker Jupiter-mCherry (Fig. 9). Beads bound to WT Spd-2-ALFA recruited all these proteins, but beads bound to Spd-2∆Polo-ALFA recruited almost no Polo-GFP or RFP-Cnn but normal (or even slightly increased) levels of AurA-GFP and mCherry-TACC, and they organized robust MTs. The TACC scaffold organized by these beads appeared less coherent, often comprising lots of smaller blobs that seemed to be being pulled outward on the bead MTs. This suggests that the Cnn scaffold on the WT Spd-2-ALFA beads can stabilize the TACC scaffold to some extent, countering its dissipation on the MTs—as is the case at centrosomes (Fig. 1, A and D). Nevertheless, beads that cannot recruit a robust Polo/Cnn scaffold can still recruit an AurA/TACC scaffold and organize a relatively robust array of MTs.

The binding of AurA-GFP and RFP-TACC was strongly perturbed on Spd-2∆ABD1 and Spd-2∆ABD2 beads, suggesting that both of these regions can contribute to AurA recruitment (Fig. 9, A and B). In agreement with our earlier experiments at centrosomes (Fig. 5C), the deletion of ABD2 had a stronger effect, and we consistently observed a very small amount of AurA being recruited to the Spd-2∆ABD1, but not Spd-2∆ABD2, beads (Fig. 9A). Potentially related to this, the recruitment of Cnn and Polo was much more perturbed by the deletion of ABD2 than ABD1, and this was also the case for MT recruitment (Fig. 9B). This suggests that the binding of AurA to Spd-2-ABD2 may play some part in allowing Spd-2 to recruit and/or activate Polo. Regardless of this difference, these findings demonstrate that Spd-2 beads can still recruit some Polo/Cnn scaffold and organize some MTs even when the recruitment of the AurA/TACC scaffold is strongly perturbed.

Beads bound to forms of Spd-2 that could not recruit Polo or AurA (Spd-2∆Polo-∆ABD1 or Spd-2∆Polo-∆ABD2) organized very few MTs (Fig. 9B), although we consistently observed that some Spd-2∆Polo-∆ABD1 beads could still recruit a small amount of AurA and organize a small amount of MTs (Fig. 9A). Together, these observations support our proposed pathway (Fig. 8A) and indicate that the ability of Spd-2 to recruit Polo and AurA is essential to drive the assembly of the Cnn and TACC scaffolds, respectively. Moreover, they demonstrate that both pathways can organize some PCM and MTs independently of each other, at least in this bead assay.

## DISCUSSION

In vertebrates, centrosomal CEP192/Spd-2 proteins were known to recruit and activate both PLK1/Polo and AURKA/AurA (*45*, *46*), playing a crucial part in centrosome and spindle pole function—although the precise roles of these proteins were unknown (*71*, *83*). In flies and worms, it was clear that Spd-2/SPD-2 recruited Polo/PLK-1 to centrosomes (*27*, *28*, *55*) to promote the assembly of a Cnn/SPD-5 mitotic PCM scaffold (*30*, *31*, *33*, *36*), but it was not known if Spd-2/SPD-2 also recruited AurA (*71*). Here, we show that *Drosophila* Spd-2 does recruit AurA, and this functions to stimulate the assembly of a second, TACC-dependent, mitotic PCM scaffold. Thus, the Spd-2/CEP192–dependent recruitment of Polo/PLK1 and AurA/AURKA to centrosomes is conserved in humans and flies, and, in flies at least, this recruitment serves to drive the assembly of two mitotic-PCM scaffolds around the centrioles.

The centrosomal TACC scaffold we describe here in flies appears to be similar to the LISD found in acentrosomal mouse oocyte meiotic spindles (*52*). Both structures rely for their assembly on the AurA-dependent phosphorylation of TACC/TACC3 (*52*, *59*, *60*) which stimulates TACC’s interaction with CHC (*67*–*69*), with CHC also being required for scaffold assembly. The LISD was originally thought to be unique to acentrosomal meiotic spindles because no similar structure was detected on mitotic spindles artificially induced to lack centrosomes (*52*). Our data suggest, however, that, on mitotic spindles, centrosomes are required to stimulate TACC scaffold assembly because the centrioles act as a source of Spd-2/CEP192. This may explain why acentrosomal mitotic spindles do not assemble an LISD. We note that CEP192 is present at acentriolar mouse oocyte spindle poles (*52*), but whether it recruits AurA to promote LISD assembly remains untested.

The biophysical nature of the mitotic PCM, and particularly the hypothesis that LLPS is crucial for its assembly, is hotly debated (*16*–*20*). Our discovery that *Drosophila* centrioles generate an AurA/TACC scaffold with liquid-like properties could be interpreted as support for the LLPS hypothesis. The disordered N-terminal region of mouse TACC3 can undergo LLPS in vitro, and the N-terminal region of *Drosophila* TACC is also predicted to be disordered—although *C. elegans* TAC-1 lacks this region (*84*). However, the observation that purified proteins in simple media can undergo LLPS is not strong evidence that LLPS is necessarily occurring in the more complicated cellular environment (*85*). Moreover, similar to the Cnn scaffold (*36*), TACC scaffold assembly seems to rely on a relatively strong stereo-specific interaction between TACC and CHC for its assembly (*86*), interactions that are not typical of LLPS. Our experiments show that molecules in the Cnn scaffold exhibit limited internal rearrangement, while those in the TACC scaffold rearrange more easily (implying a more liquid-like behaviour). We think it is possible, however, that both scaffolds may best be described as porous viscoelastic gels (albeit with different viscoelastic properties) that are permeated by the cytoplasm. As argued previously, experimentally testing whether the assembly of either scaffold is driven by LLPS in vivo will be challenging (*18*, *87*).

Regardless of their assembly mechanism, both scaffolds are required for proper centrosome assembly in fly embryos, although our bead-reconstitution experiments indicate that each can independently recruit PCM and organize microtubules, at least to some extent. Clearly, in addition to its role in directly helping to recruit at least some PCM-client proteins (*61*, *62*, *88*, *89*), the solid-like Cnn scaffold also provides overall mechanical strength to the PCM (*90*), but the reasons why the more liquid-like properties of the TACC scaffold may be required are less obvious. One possibility is that the liquid-like behavior of the TACC scaffold allows it to expand more easily around the centrioles to form a more extensive “net” around the centrioles, allowing the scaffold to capture centriole and centrosome proteins over a large volume and increasing their local concentration around the centriole. In support of this possibility, the outward flux of Spd-2 molecules from the centriole that we demonstrate here seems to be required for the outward expansion of the solid-like Cnn scaffold, but not for the expansion of the more liquid-like TACC scaffold.

It is currently unknown whether TACC proteins form a scaffold in systems other than the fly embryo and mouse oocyte, although a TACC scaffold was observed in other mammalian female meiotic spindles (*52*). The AurA-dependent phosphorylation of TACC that drives its interaction with CHC, and so TACC scaffold assembly, is conserved and has been shown to occur in cultured human and chicken somatic cells (*86*, *91*), indicating that a TACC scaffold could potentially assemble in non-ooctye/embryo systems. Intriguingly, TACC3 in human cells (*53*), the LISD in mouse oocytes (*52*), and the TACC scaffold in fly embryos (*65*), all spread out extensively around the centrosome/spindle poles, suggesting that the ability of these proteins to form an extended structure is a conserved feature of their function.

The pathway outlined in Fig. 8A can explain why mother centrioles are normally the dominant sites of mitotic PCM assembly, as they provide a source of Spd-2/CEP192. Our bead-reconstitution experiments demonstrate the importance of Spd-2, as simply concentrating Cnn or TACC on the bead surface is not sufficient to initiate PCM assembly, even when the embryo is in mitosis and Polo and AurA activity in the cytoplasm are presumably high. This is probably because binding to Spd-2 is required to activate Polo and AurA in such a way that they can phosphorylate Cnn and TACC, respectively, to initiate scaffold assembly. The N-terminal 1000 amino acids of *Xenopus* CEP192 can also organize microtubules when recruited to synthetic beads in mitotic extracts of *Xenopus* eggs, and this is dependent on AurA/AURKA and/or Polo/PLK1 recruitment (*45*). Thus, we propose that Spd-2/CEP192, Polo/PLK1, and AurA/AURKA may form a conserved nexus that drives mitotic centrosome assembly in most, if not all, cell types and species that build mitotic centrosomes. However, the relative contribution of the Polo/Cnn and AurA/TACC scaffolds, the level of their interdependency, and the precise downstream clients they recruit will likely vary between cell types and species.

## MATERIALS AND METHODS

### *Drosophila melanogaster* stocks and husbandry

The *Drosophila* stocks used, generated and/or tested in this study are listed in table S1. The precise stocks used in each experiment (and the relevant figure) are listed in table S2. Flies were maintained on *Drosophila* culture medium (0.68% agar, 2.5% yeast extract, 6.25% cornmeal, 3.75% molasses, 0.42% propionic acid, 0.14% tegosept, and 0.7% ethanol) in 8 cm–by–2.5 cm plastic vials or 0.25-pint plastic bottles. For microscopy and immunoblot experiments, flies were placed in embryo collection cages on fruit juice plates (see below) with a drop of yeast paste. Fly handling was performed as previously described (*92*).

### Transgenic fly line generation

Transgenic fly lines were generated via random P-element insertion (injected, mapped, and balanced by The University of Cambridge Department of Genetics Fly Facility). For transgene selection, the *w^+^* gene marker was included in the transformation vectors, and constructs were injected into the *w^1118^* genetic background.

### Molecular biology

To generate NG-TACC transgenic flies, cDNA fragment encoding TACC was amplified using primers s1 and s2 (table S3) containing the attB sites and transferred to a pDONR plasmid backbone using a BP clonase (Gateway Technology, Thermo Fisher Scientific) to generate a pDONR-TACC. For non-phosphorylatable mutant TACC-S863L, primers s3 and s4 (table S3) were used to create a single amino acid mutation on the pDONR-TACC. Flipping TACC or TACC-S863L from pDONR vector to pUbq-mNeonGreen (NG) or pUbq-mCherry destination vector using a LR clonase (Gateway Technology, Thermo Fisher Scientific), an N terminally fused pUbq-NG-TACC or pUbq-NG-TACC-S863L or pUbq-mCherry-TACC or pUbq-mCherry-TACC-S863L plasmids, was generated. We note that serine originally designated as Ser^863^ in this previous paper is Ser^900^ in the 1227–amino acid long TACC-PA isoform listed in FlyBase, which is the isoform we use in the experiments reported here. To generate Ubq-Klp10A-GFP, an entry vector containing Klp10A without stop codon (pENTR4-Klp10A_LD29208 cDNA) was obtained (*93*). Klp10A was then introduced into pUbq-mGFP destination vector using LR clonase (Gateway Technology, Thermo Fisher Scientific).

To generate Spd-2-GFP, GFP-Cnn, and GFP-TACC constructs for in vitro mRNA synthesis, pDONR vectors containing Spd-2, Cnn, or TACC CDS were recombined with a destination pRNA vector containing a GFP CDS at either the N or C terminus using Gateway Technology (Thermo Fisher Scientific).

For various Spd-2 variants fused to mScarlet-I3 (*74*), a mScarlet-I3 (I3) expression vector was purchased from Addgene (#189755) and later optimized to *Drosophila* codon usage by Genewiz. The pRNA destination backbone was linearized by primers s5 and s6 (table S3) and assembled with the mScarlet-I3 fragment using a NEBuilder HiFi DNA Assembly kit (NEB) to create a pRNA-I3 destination vector. pDonor vector containing Spd-2 cDNA was flipped into pRNA-mScarlet-I3 destination vector using LR clonase (Gateway Technology, Thermo Fisher Scientific) to generate pRNA-Spd-2-mScarlet-I3. Similarly, a pDonor containing Spd-2∆Polo (previously known as Spd-2-ALL) was flipped to generate a pRNA–Spd-2-∆Polo-mScarlet-I3. For Spd-2∆ABD1 and Spd-2∆ABD2, the targeted sequences in pRNA-Spd-2-I3 and pRNA-Spd-2-GFP were removed by site-directed mutagenesis using either primers s7 and s8, or primers s9 and s10 (table S3) to generate linearized pRNA-Spd-2∆ABD1-I3, pRNA-Spd-2∆ABD1-GFP, pRNA-Spd-2∆ABD2-I3 and pRNA-Spd-2∆ABD2-GFP, respectively. pRNA-Spd-2∆ABD1-GFP and pRNA–Spd-2∆ABD2-GFP were linearized by primers s11 and s12 (Supplementary Table 3) to introduce an ALFA sequence followed by a stop codon in the C terminus of Spd-2, which can be efficiently recognized by anti-ALFA nanobody (*82*). To generate Spd-2∆Polo-∆ABD2 and Spd-2∆Polo-∆ABD2, pRNA-Spd-2∆Polo-ALFA plasmid was linearized by primers either s7 and s8 or s9 and s10 (table S3) and circularized using a KLD Enzyme Mix (M0554S, NEB).

For Spd-2-SNAP, a pDONR vector containing a full-length Spd-2 cDNA was used. pSNAPf vector containing SNAP-tag was purchased from NEB (N9183S) and optimized to *Drosophila* codon suing Genewiz (USA), which was subcloned to make a pUbq-SNAP destination vector using primers s13 and s14 (table S3), which contain regions from a pUbq destination vector. The amplified fragment containing SNAP was assembled with an amplified pUbq destination backbone using a NEBuilder HiFi DNA Assembly kit (NEB) to create a pUbq-SNAP destination vector. Spd-2 from a pDONR vector was introduced into pUbq-SNAP destination vector using LR clonase (Gateway Technology, Thermo Fisher Scientific).

To generate expression plasmid for anti-ALFA nanobody, anti-ALFA nanobody fragment was amplified using primers s15 and s16 (table S3) from its expression vector (Addgene, #189755). A pET24a-VHH-std vector (Addgene, #109417) was linearized by primers s17 and s18 (tableS3) that overlapped the sequence of anti-ALFA nanobody. These fragments were assembled using a NEBuilder HiFi DNA Assembly kit (NEB) to create a pET24a-anti-ALFA–std expression vector. A complete list of the primers and plasmids used or generated in this study are listed in tables S3 and S4.

### Nanobody-coated beads preparation

A 0.1 mg of Dynabeads MyOne Streptavidin C1 (65001, Invitrogen) was washed 3× in phosphate-buffered saline (PBS) supplemented with 0.01% Triton X-100 (T9284, Sigma-Aldrich) and 0.1% bovine serum albumin (A7906, Sigma-Aldrich) (PBSTB). The beads were incubated with either biotinylated anti-GFP or anti-ALFA nanobody (4.4 μg/ml—prepared as described below) in PBSTB for a minimum of 30 min at room temperature. The beads were then washed 3× in PBSTB and stored at a concentration of 3 mg/ml at 4°C until further use (for maximum efficiency, the beads were used within 1 week after preparation). For embryo injection, the nanobody-coated beads were diluted with either mRNA solution or water to achieve a final concentration of 1 mg/ml.

### Nanobody expression and purification

Recombinant anti-GFP (*94*) or anti-ALFA-tag (*82*) nanobody was cotransformed with pET-21d-myc-BirA (to biotinylate the Nanobody) (Addgene, #109424) into Rosetta (DE3) bacterial cells. Cells were grown on a shaking incubator using the appropriate antibiotics and 200 μM dbiotin in LB broth at 37°C until an optical density at 600 of 0.6 to 0.8 was reached. Protein expression was then induced with 1 mM isopropyl β-d-1-thiogalactopyranoside, and cells were grown overnight at 18°C. Cells were pelleted, washed, resuspended in binding PBS buffer supplemented with Complete EDTA-free Protease inhibitor Cocktail (Roche), and lysed using an Emulsiflex-C5 homogenizer (Avestin). The soluble protein fraction was then purified using a HisTrap HP prepacked column (Cytiva). An extra purification step of size exclusion chromatography was carried out using an S75 16/600 column (GE healthcare) equilibrated against a PBS buffer pH 7.5. Purified anti-GFP or anti-ALFA-tag nanobodies were stored in liquid nitrogen at ~1 mg/ml.

### mRNA synthesis

For in vitro transcription, the constructs were digested and linearized by Asc I (R0558S, NEB) and precipitated with 10 mM sodium acetate and 7 mM EDTA in 66% ethanol overnight at −20°C. Precipitated DNA was washed with 70% ethanol, dried, and subsequently dissolved in diethyl pyrocarbonate–treated water (AM9906, Ambion). Around 1.6 to 3.2 μg of digested DNA were used to synthesize mRNA with the T3 mMESSAGE mMACHINE Kit (AM1348, Thermo Fisher Scientific). The mRNA product was purified with the RNeasy MinElute Cleanup Kit (74204, QIAGEN). All RNAs were stored at −70°C. The final concentrations of RNA used in various experiments are described in the next section.

### Embryo collection and injection

Embryos were collected from plates (40% cranberry-raspberry juice, 2% sucrose, and 1.8% agar) supplemented with fresh yeast suspension. For live-imaging experiments, embryos were collected for 1 hour at 25°C and aged at 25°C for 45 to 60 min. Embryos were dechorionated by hand, mounted on a strip of glue on either a 35-mm glass-bottom petri dish with 14-mm micro-well (MatTek) or high precision 35-mm high glass bottom μ-dishes (ibidi; for FCS experiments), and desiccated for 1 min at 25°C before covering with Voltalef oil (H10S PCTFE, Arkema) to avoid further desiccation.

For colchicine injection, embryos were desiccated for 5 to 10 min (depending on the ambient conditions) before covering with Voltalef oil and injecting colchicine [100 μg/ml; diluted in Schneider’s medium from a stock (1 mg/ml) in dimethyl sulfoxide (DMSO)]. Embryos were imaged on the PerkinElmer spinning disk system described below.

For single-molecule experiments, embryos were aged at 25°C for 30 min and desiccated for 5 min. After covering with Voltalef oil, they were injected with SNAP dye JF549 (GA1110, Promega), dissolved in DMSO (*77*) at concentrations ranging from 1 to 1000 nM, and incubated for a further 30 min at 25°C before imaging on the Andor DragonFly 505 (Oxford Instruments) spinning disk system described below.

For synthetic bead injection, nanobody-coated beads were mixed with mRNA or water to achieve a final concentration of 1 mg/ml for beads and 150 μg/ml for mRNA. Embryos were collected for 30 min at 25°C, dechorionated, and desiccated for 5 min. After covering with Voltalef oil, embryos were injected with the nanobody-coated beads and mRNA mix and incubated for a further 30 min at 25°C before imaging on the Andor DragonFly 505 (Oxford Instruments) spinning disk system described below.

For mRNA only injection, mRNA was diluted to a final concentration of 2 mg/ml. Embryos were collected for 30 min at 25°C, dechorionated, and desiccated for 5 min. After covering with Voltalef oil, embryos were injected with the nanobody-coated beads and mRNA mix and incubated for a further 1 hour at 22°C before imaging on the Andor DragonFly 505 (Oxford Instruments) spinning disk system described below.

### Spinning disk confocal microscopy

Images of living embryos were acquired at 23°C using a PerkinElmer ERS spinning disk confocal system mounted on a Zeiss Axiovert 200M microscope using Volocity software (PerkinElmer). A 63×, 1.4 numerical aperture (NA) oil objective was used for all acquisition. The oil objective was covered with an immersion oil (ImmersolT 518 F, Carl Zeiss) with a refractive index of 1.518 to minimize spherical aberration. The detector used was a charge-coupled device (CCD) camera (Orca ER, Hamamatsu Photonics, 15 bit), with a gain of 200 V. The system was equipped with 405-, 488-, 561-, and 642-nm solid-state lasers (Oxxius S.A.). The microscope was operated using a Volocity software. All red/green fluorescently tagged samples were acquired using UltraVIEW ERS “Emission Discrimination” setting. The emission filters used were a green long-pass 520-nm emission filter and a red long-pass 620-nm emission filter. For dual channel imaging, the red channel was imaged before the green channel in every slice in a z-stacks. A total of 0.5-μm *z* sections were acquired, with the number of sections, time step, laser power, and exposure depending on the experiment. For FRAP experiments, multiple circular regions of interests with a diameter of 3.5 to 4 μm were created around different centrosomes. Lasers (488 nm and 521 nm) with 50% laser power of 20 iterations were used to FRAP each sample. In some samples, different centrosomes were bleached at different time points in the same embryos. *z* sections (0.5 μm) were acquired, with the number of sections, time step, laser power, and exposure depending on the experiment.

For single molecule, synthetic bead, and mRNA injection experiments, embryos were imaged on an Andor Dragonfly 505 (Oxford Instruments) spinning disk confocal microscope (40-μm pinhole size), which was mounted on a Leica DMi8 stand, using Fusion software. Solid-state diode lasers (561 and 488 nm) were used to image JF549 and mNG, respectively, using a 63×/1.40NA oil immersion objective and an Andor iXon Ultra 888 electron multiplying CCD camera. Stacks consisting of eight slices with a *z* spacing of 0.5 μm were acquired every 10 s for 30-min total duration for single-molecule tracking. For synthetic bead analysis, a single stack consisting of 41 slices with a *z* spacing of 0.5 μm was collected for each embryo. For mRNA injection experiments, an Andor Sona complementary metal-oxide semiconductor camera was used to image embryos injected with various Spd-2 variants. A single stack consisting of 17 slices with a *z* spacing of 0.5 μm was collected for each embryo.

### Super-resolution spinning disk confocal microscopy

Super-resolution imaging was performed using a SoRA disk (Yokagawa), a 3.2× magnification lens (Olympus), and a photometrics BSI camera (95% QE, 6.5 μm pixels) mounted on an Olympus IX83 microscope equipped with a 60×/1.3 NA silicon immersion lens. The oil objective was covered with an immersion oil (ImmersolT 518 F, Carl Zeiss) with a refractive index of 1.518 to minimize spherical aberration. Samples were excited with QBIS 488- or 561-nm laser (Coherant) with 525/50- or 617/75-nm emission bandpass filters, respectively. A “quad” dichoric of 405/488/561/640 nm was used. At least six *z* sections with 0.5 μm in thickness each were used for each image. Laser power and exposure were adjusted according to the experiment.

### Fluorescence correlation spectroscopy

Point FCS measurements were performed and analyzed as previously described (*64*). All measurements were conducted on a confocal Zeiss LSM 880 [argon laser excitation at 488 nm and GaAsP photon-counting detector (491 to 544 nm detector range)] with Zen Black Software. A C-Apochromat 40×/1.2 W objective and a pinhole setting of 1 Airy unit (AU) were used, and spherical aberrations were corrected for on the correction collar of the objective at the beginning of each experimental day by maximizing the FCS-derived counts-per-molecule (CPM) value of a fluorescent dye solution. The effective volume Veff of this system was previously estimated to be ∼0.25 fl (*95*). Measurements were conducted with a laser power of 6.31 μW, and no photobleaching was observed for any protein. The temperature of the microscope was kept between 25.0° and 26.0°C using the Zeiss inbuilt heating unit XL. For experimental FCS recordings, consecutive cytoplasmic measurements were made 6× for 4 s each at the centrosomal plane of the embryo. For measurements within the PCM, a snapshot of the centrosomes in the embryo was taken before initiating the FCS measurements, and at the end of the FCS measurements, and the data were discarded if the centrosome had moved away from the measurement point. In addition, any erratic autocorrelation functions from the cytoplasmic measurements (usually generated when a centrosome or yolk granule moved into the point of measurement) were also discarded. All remaining curves were then fitted with eight different diffusion models in the FoCuS-point software, including one or two diffusing species with no dark state of the fluorophore, one dark state of the fluorophore (either triplet or blinking state), or two dark states of the fluorophore (triplet and blinking state).

### Image data analysis

For centrosome analysis, raw images were *z*-projected using the maximum intensity projection function, and the background was estimated and corrected using an uneven illumination background correction using a custom Python script. Centrosomes were detected and located by a Crocker-Grier centroid-finding algorithm (*96*) available in the Python library TrackPy (*97*). The signal-to-background intensity threshold was calculated by an Otsu algorithm (*98*). The sum intensity and area of each centrosome were calculated from the segmented region based on the Otsu threshold.

For FRAP analysis, raw time-series images were corrected for photobleaching using the exponential decay function, *z*-projected using the maximum intensity projection function, and the background was estimated and corrected using an uneven illumination background correction (*99*). Photobleached centrosomes were manually tracked using a software package developed for mobile robotics (*100*). A custom Python script was used to extract the fluorescence intensities at tracked centrosomes as they changed over time in each individual embryo, as previously described (*101*).

Synthetic bead fluorescence intensity data were analyzed using Fiji (ImageJ2 ver.2.3.0/1.53q). Raw single-frame images were *z*-projected using the maximum intensity projection function, and background was corrected using an uneven illumination background correction. GFP beads were auto-selected, and their intensities were calculated by the TrackMate plugin (*102*) with an estimated object diameter of 2.7 μm. The fluorescence mean intensity of each bead was measured and analyzed using scripts generated by ChatGPT4 before visualization using GraphPad Prism.

The single-molecule Spd-2–NG data were analyzed using Fiji. Hyperstacks corresponding to time-lapse videos of embryos were maximum intensity projected onto a single *z* plane and bleach corrected. Trackmate (*102*) was used to detect and track single particles of JF549 covalently bound to SNAP-tagged Spd-2. Single molecules were defined with an estimated object diameter of 0.5 μm in the orange channel with a LoG detector and were tracked over time using a simple linear assignment problem (LAP) tracker, with linking max distance of 2.0 μm, gap-closing max distance of 2.0 μm, and gap-closing max frame gap of 1. Each tracked particle was inspected for colocalization with the mother centriole—recognized with the Asl-NG marker (*78*). The following rules were used as criteria to select single-molecule–binding events: (i) Only embryos with sparse labeling (<5% centrosomes labeled at any one time point) were analyzed; (ii) incomplete tracks, in which we did not detect the molecule entering and/or leaving the centrosome, were excluded; (iii) tracks in which molecules were only observed at the centrosome for one time point were excluded; (iv) we included tracks where molecules disappeared for a single timepoint but then reappeared in the next as single fluorophores can blink into temporary dark states (*103*); (v) the occasional centrosomes where more than one binding event occurred at the same time were excluded. Mother centrioles with JF549 particle tracks that met these criteria were then isolated and centered, and a custom Python script was used to calculate the distance between the center of mass of the centriole (in the green channel) and the brightest pixel of the JF549 particle (in the orange channel) at successive time points.

SoRA super-resolution images were enhanced using Olympus Super-Resolution (OSR) algorithm with a low filtering strength and subsequently deconvolved using a constrained iterative deconvolution algorithm with five iterations in CellSense software (Olympus).

### Protein structure prediction and visualization

We initially screened for potential interactions between *Drosophila* AurA and Spd-2 using ColabFold v1.4.0 (*104*) in which AlphaFold2-multimer V2 was embedded to predict potential interaction interfaces between the full-length proteins and/or various fragments of the proteins. Automatic model type was specified in run setup with number of recycles defined to three with no template information used. Using the iPTM score as an initial assessment of each predicted interaction, we identified the interaction between the AurA kinase domain (AurA_155-421_) and Spd-2_291–310_ (subsequently named ABD2) as the strongest hit (iPTM = 0.769). Shortly afterward, a potential interaction interface between HsAURKA and HsCEP192 was identified (*70*). This was different to the potential interaction we initially identified, but in a screen using ColabFold v1.5.5 in which AlphaFold2-multimer V3 was embedded where we used the N-terminal half of Spd-2 (Spd-2_1-650_) with the AurA kinase domain (AurA_155-421_), an interaction interface similar to the one identified in humans was found with Spd-2_229-250_ (later named ABD1). Subsequent studies suggested that both ABD1 and ABD2 contributed to the recruitment of AurA to centrosomes, so we used AlphaFold2 ColabFold to predict the interaction interface between the AurA kinase domain and a combined Spd-2 peptide that contained both ABD1 and ABD2 (AurA_155-421_ and Spd-2_229-310_) (iPTM = 0.59). PDB files for the human AURKA-Cep192 (PDB ID:8GUW) and AURKA-TPX2 (PDB ID: 5ODT) structures were downloaded from previously published structures on the PDB. UCSF Chimera X-1.7.1 (*105*) was used for structural analysis and figure generation.

### Immunoblotting

Embryos for immunoblotting were fixed and stored in methanol as described previously (*106*). Afterward, the embryos were stored at 4°C at least overnight and rehydrated with 3× PBT (PBS + 0.1% Triton X-100) washes for 15 min each before being subjected to electrophoresis and immunoblotting as described previously (*95*). The following primary antibodies were used: mouse anti-GFP (#11814460001, Roche), rabbit anti-TACC (1:1000) (*54*), rabbit anti-GAGA factor (1:500) (*107*), mouse anti-actin (A3853, Sigma-Aldrich), and rabbit anti-GFP (A6455, Molecular Probes). Horseradish peroxidase–conjugated donkey antirabbit (NA934V) and antimouse [NA931-1 M (both from VWR International Ltd.)] secondary antibodies were used at 1:3000 to 1:5000. Diluted SuperSignal ECL substrates (Thermo Fisher Scientific) were used to develop chemiluminescence signal.

### Statistical analysis

The details of statistical tests, sample size, and definition of the center and dispersion are provided in individual figure legends.

## Supplementary Materials

### The PDF file includes:

Figs. S1 to S6 Legends for movies S1 to S6 Tables S1 to S4 References
