# Supplementary material for: Centrioles generate two scaffolds with distinct biophysical properties to build mitotic centrosomes
Source: Sci Adv. 2025 Feb 7;11(6):eadq9549. doi: 10.1126/sciadv.adq9549 (PMC11804907; doi:10.1126/sciadv.adq9549)
Supplement: Supplementary file 1 — Figs. S1 to S6 Legends for movies S1 to S6 Tables S1 to S4 References [file sciadv.adq9549_sm.pdf]

Science Advances
AAAS

Supplementary Materials for

Centrioles generate two scaffolds with distinct biophysical properties to build mitotic centrosomes

Siu-Shing Wong *et al.*

Corresponding author: Siu-Shing Wong, isaac.wong@path.ox.ac.uk; Jordan W. Raff, jordan.raff@path.ox.ac.uk

Sci. Adv. **11**, eadq9549 (2025)
DOI: 10.1126/sciadv.adq9549

The PDF file includes:

Figs. S1 to S6
Legends for movies S1 to S6
Tables S1 to S4
References

Other Supplementary Material for this manuscript includes the following:

Movies S1 to S6

Fig. S1

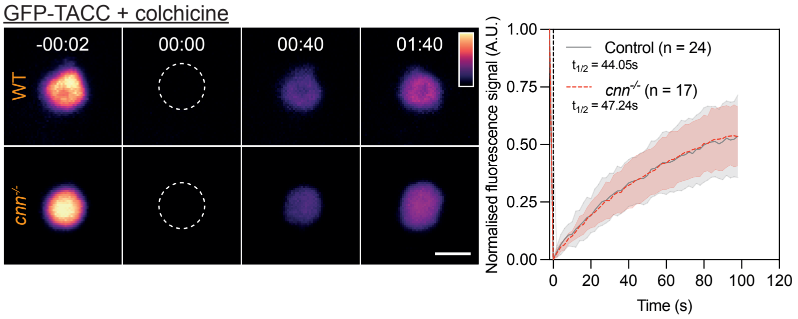

Fig. S1 GFP-TACC recruitment to centrosomes does not appear to depend on Cnn. Images show, and graph quantifies, the centrosomal-fluorescence recovery (Mean±SD) after photobleaching of GFP-TACC in WT or *cnn*$^{-/-}$ embryos treated with colchicine. Time (mins:secs) is indicated; centrosomes were bleached at t=0:00. $t_{1/2}$ was calculated from a fitted One-Phase Association Model (Graphad Prism). N=10-15 embryos, n=17-24 centrosomes. Scale bar = 2µm.

Fig. S2

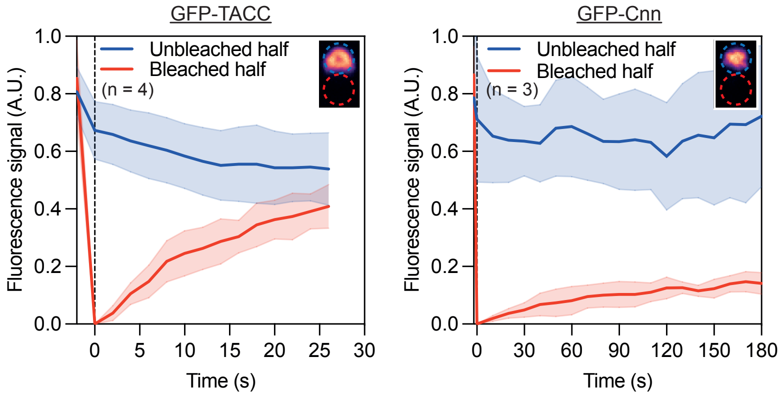

Fig. S2 Quantification of partial FRAP of closely-paired centrosomes labelled with GFP-TACC or GFP-Cnn. Graphs quantify the centrosome-fluorescence (Mean±SD) of the unbleached centrosome (blue lines) and the bleached centrosome (red lines) in centrosome pairs from colchicine-injected embryos expressing either GFP-TACC (n = 4 centrosome pairs) or GFP-Cnn (n = 3 centrosome pairs). Note how the fluorescence of the unbleached centrosome in GFP-TACC expressing embryos decreases, while in embryos expressing GFP-Cnn it remains relatively constant. This indicates that the bleached molecules can readily move towards the unbleached centrosome (so decreasing its fluorescence) in the case of GFP-TACC expressing embryos, but not GFP-Cnn expressing embryos.

Fig. S3

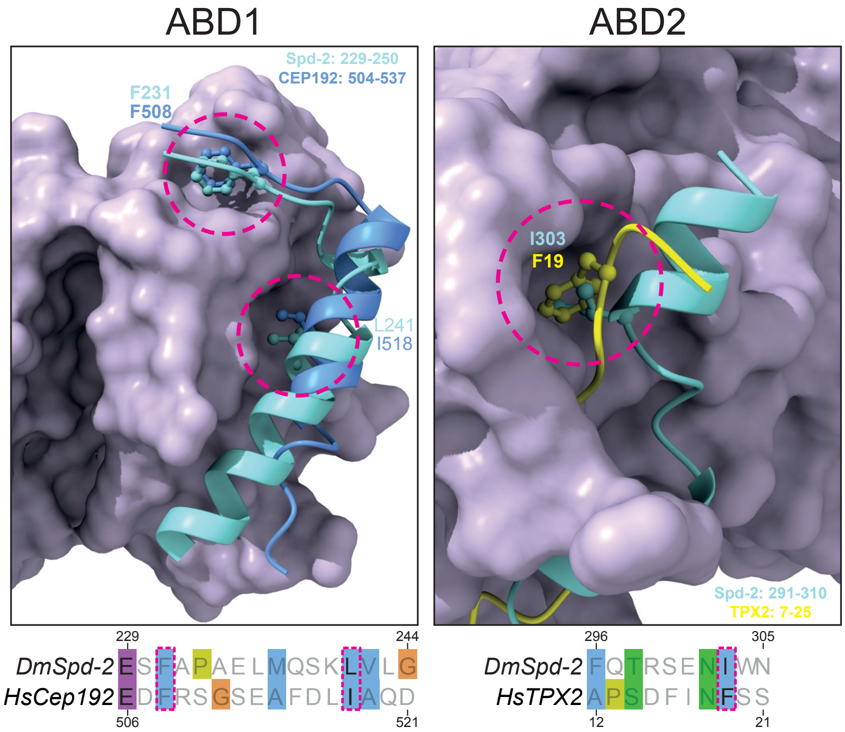

Fig. S3 Analysis of the predicted structure of the Spd-2 AurA binding sites (ABD1 and ABD2). Images show a detailed view of the predicted interaction interface between the AurA kinase domain (*magenta*) and either ABD1 (*cyan*, left box) or ABD2 (*cyan*, right box), together with the crystal structure of the previously characterised region of either human CEP192 (*blue*, left box) or human TPX2 (*yellow*, right box) interacting with AURKA (but both shown here overlayed on the *Dm*AurA structure). The sequences of the protein regions that interact with the AurA/AURKA kinase domain are aligned underneath the images (coloured using the Clustal Omega scheme). These sequences show limited conservation, but aspects of the binding of *Dm*Spd-2-ABD1 and *Hs*CEP192 to AurA/AURKA appear similar, and the position of two conserved hydrophobic amino acids that insert into the AurA/AURKA binding regions are highlighted with *red-dotted lines*. Interestingly, the substitution of the equivalent hydrophobic residues (F629 and I639) with charged Arg in *Xenopus* CEP192 strongly inhibits AurA binding *(40)*, and an Alanine substitution of the equivalent Phe in *Drosophila* Spd-2 (F231A) also strongly perturbs Spd-2's ability to recruit AurA to synthetic beads (see B,C, below), supporting the likely accuracy of the predicted Drosophila structure. Interestingly, human TACC3 also interacts with this region of the AurA kinase domain *(67)*, with TACC3 F525 binding in a similar manner to F231 and F508 in the fly and human Spd-2/CEP192 proteins (not shown). Although *Dm*Spd-2-ABD2 and *Hs*TPX2$_{12\text{-}21}$ clearly bind to a similar region on the AurA/AURKA surface, there is very limited similarity in how they do so—although a hydrophobic amino acid that inserts into a similar pocket on the AurA/AURKA surface is also highlighted in red-dotted lines. We have not yet investigated the potential significance of this hydrophobic amino acid for the AurA interaction.

Fig. S4

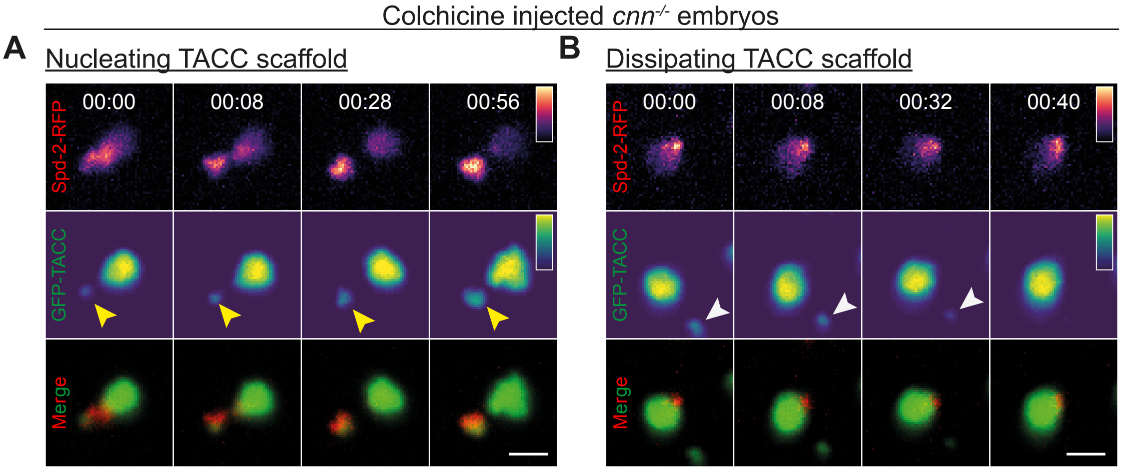

Fig. S4 Centrioles appear to be required to generate the TACC scaffold. (A,B) Images from time-lapse movies show centrosomes in colchicine-injected *cnn*$^{-/-}$ embryos co-expressing Spd-2-RFP and GFP-TACC. Time (mins:secs) is indicated. In **(A)** the centriole (*yellow arrowhead*) becomes separated from the main bulk of the TACC scaffold. New TACC scaffold starts to accumulate around the centriole. The centriole-less TACC scaffold remains stable over this short timescale, but will eventually dissipate (not shown). **(B)** This dissipation can be appreciated more easily at the centrosome shown here, where a smaller "flare" of GFP-TACC that lacks a centriole (*white arrowhead*) has become separated from the main TACC scaffold associated with the centriole. The flare lacks detectable Spd-2-RFP and it quickly dissipates. Scale bars = 2µm.

Fig. S5

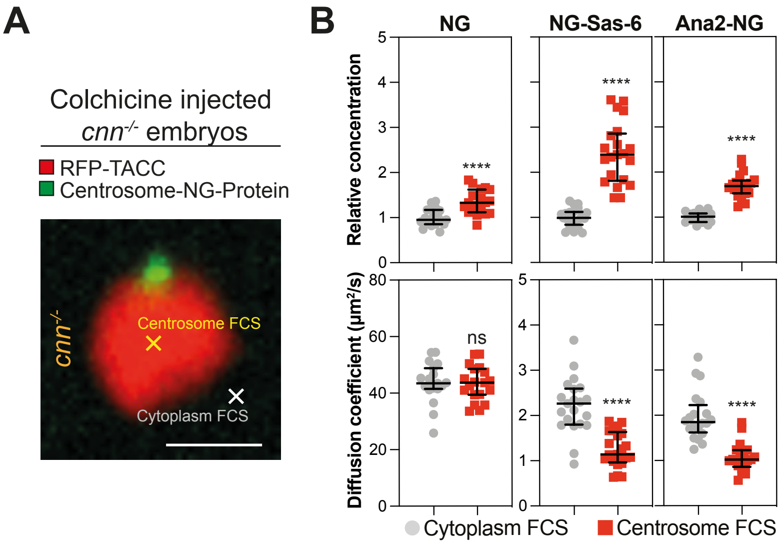

<u>Fig. S5 FCS comparison of protein behaviour at centrosomes in *cnn-/-* embryos.</u> **(A)** Image shows a centrosome in an embryo expressing RFP-TACC and Ana2-NG and injected with colchicine. This illustrates the typical areas that were analysed by FCS in the outer regions of the centrosome (yellow cross) or the nearby cytoplasm (white cross). In the absence of Cnn scaffold, the PCM appears to be structurally weakened and the centriole cannot maintain its position at the centre the PCM *(90)*. Scale bar = 2μm. **(B)** Scatter plots show the FCS-measured concentration (top plots) or diffusion rate (bottom plots) (Median±Quartiles) of NG or various NG-fusions in the cytoplasm (grey circles) or in the outer regions of the centrosome (red squares) in WT embryos injected with colchicine. N=20-25 embryos. Statistical significance was calculated using Mann-Whitney's test (****: P<0.0001, ns: not significant).

Fig. S6

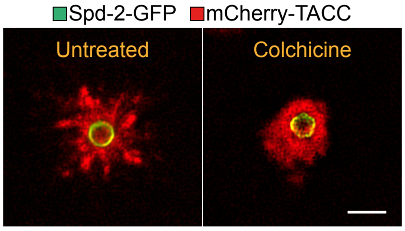

Fig. S6 The TACC scaffold generated at synthetic beads behaves similary to that generated at endogenous centrosomes. Images show Spd-2-GFP (*green*) coupled beads recruiting mCherry-TACC (*red*) in either untreated embryos or in embryos injected with colchicine to depolymerise the MTs. Note how the TACC scaffold appears to be being pulled out along the MTs organised by the bead in the untreated embryos, but not in embryos treated with colchicine. Thus, although the TACC scaffold can be pulled outwards along the bead-organised MTs, it is not dependent on these forces to drive the expansion of the scaffold around the bead. Scale bars = 2μm.

Movie S1

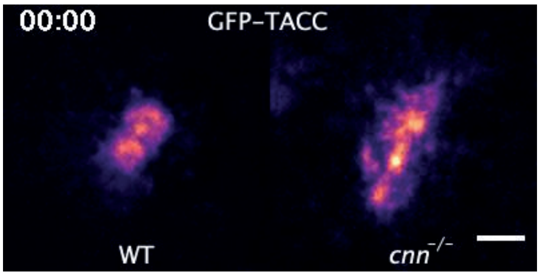

<u>Movie S1 A comparison of GFP-TACC localisation at centrosomes in WT and– *cnn*$^{-/-}$ embryos.</u> GFP-TACC is normally tightly focused at centrosomes in WT embryos (left). In the absence of Cnn (right), the PCM structure appears to be weakened *(90)* and GFP-TACC is more easily dispersed along the centrosomal MTs. Images were acquired every 30secs, and Time (mins:secs) is indicated. Scale bar = 2μm.

Movie S2

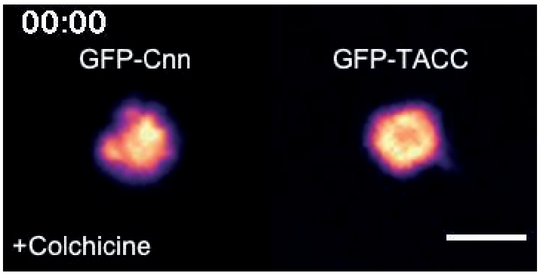

<u>Movie S2 A FRAP comparison of GFP-Cnn and GFP-TACC centro-some-dynamics.</u> Centrosomes in colchicine-injected embryos expressing either GFP-Cnn (left) or GFP-TACC (right) were bleached (t=00:02) and fluorescence recovery was monitored. GFP-Cnn recovered slowly and preferentially in the central region of the PCM around the centriole. GFP-TACC recovered more quickly and more evenly throughout the PCM volume. Images were acquired every 2secs, and Time (mins:secs) is indicated. Scale bar = 2μm.

Movie S3

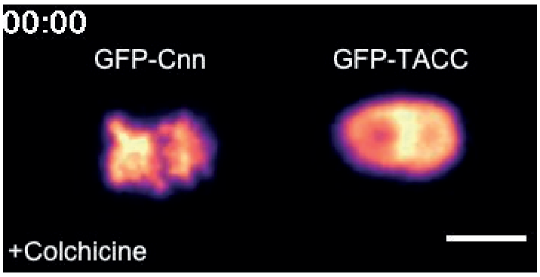

<u>Movie S3 A comparison of GFP-Cnn and GFP-TACC at closely abutted centrosome-pairs in colchicine-injected embryos.</u> A clearly demarcated boundary was usually visible between the two closely abutted centrosomes in GFP-Cnn expressing colchicine-injected embryos (left), but not in embry-os expressing GFP-TACC (right). Images were acquired every 2secs, and Time (mins:secs) is indicated. Scale bar = 2μm.

Movie S4

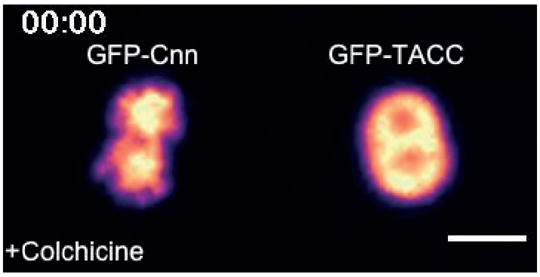

<u>Movie S4 A FRAP analysis of GFP-Cnn and GFP-TACC dynamics at closely abutted centrosome-pairs in colchicine-injected embryos.</u> One centrosome in two closely abutted centrosome-pairs in colchicine-injected embryos expressing either GFP-Cnn (left) or GFP-TACC (right) was bleached (t=00:02) and fluorescence recovery was monitored. GFP-Cnn fluorescence recovers independently around the central region of the bleached centrosome; there is no detectable movement of any unbleached GFP-Cnn molecules from the nearby centrosome into the bleached area. In contrast GFP-TACC molecules from the unbleached centrosome appear to rapidly move into the bleached centrosome region. Images were acquired every 2secs, and Time (mins:secs) is indicated. Scale bar = 2μm.

Movie S5

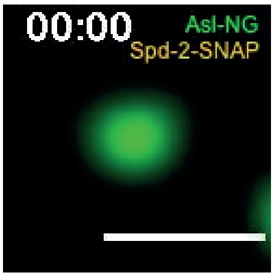

<u>Movie S5 Tracking a single particle of Spd-2-SNAP as it binds to the centriole and fluxes outwards.</u> Video shows a centrosome in an embryo expressing Asl-NG (*green*) *(95)* to label the mother centriole *(78)*, and Spd-2-SNAP, which is sparsely fluorescently-labelled by the injection of 1nM JF-549 SNAP-ligand. A single molecule of Spd-2 binds at the mother centriole and then gradually fluxes outwards away from the centriole. Time (mins:secs) is indicated. Images were acquired every 10secs, and Time (mins:secs) is indicated. Scale bar = 2μm.

Movie S6

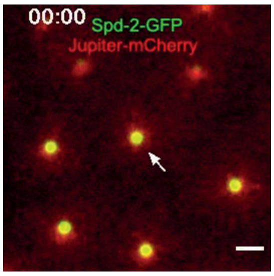

<u>Movie S6 Synthetic beads bound to Spd-2 organise MTs.</u> Movie shows a field of synthetic beads coated with an anti-GFP nanobody that have been injected into an embryo expressing Spd-2-GFP (*green*) and Jupiter-mCherry to stain the MTs (*red*). Spd-2-GFP binds the endogenous centrosomes and to the injected beads. The movie is centred around a single bead (*arrow*), highlighting how the beads organise MTs that grow and shrink (short in mitosis, long in interphase) in synchrony with the MTs organised by the endogenous centrosomes. Time (mins:secs) is indicated. Images were acquired every 30secs, and Time (mins:secs) is indicated. Scale bar = 4μm.

Supplementary Tables

Table ST1 *Drosophila* stocks used in this study

Allele	Source
cnn^{f04547}	Exelixis stock no. f04547, Exelixis Stock Centre (Harvard Medical School, Boston, MA).
cnn^{HK21}	(*109*, *110*)
GFP-Cnn	(*111*); CRISPR Knock-in fast folded GFP
Ubq-GFP-TACC	(*65*)
Ubq-NG-Cnn	(*101*)
$tacc^{stella592}$	(*58*)
aur^{1}	(*112*)
plp^{2172}	(*113*)
Spd-2^{z35711}	(*26*)
$Plk4^{Aa74}$	(*114*)
Ubq-RFP-Cnn	(*115*)
Ubq-GFP-TACC-S863L	(*65*)
Ubq-NG-TACC	Generated in this study
Ubq-NG-TACC-S863L	Generated in this study
$tacc^{59}$	(*116*); Gift from Fengwei Yu
$tacc^{74}$	(*116*); Gift from Fengwei Yu
Ubq-Aurora A-GFP	(*90*)
Ubq-Spd-2-RFP	(*34*)
Ubq-Spd-2-SNAP	Generated in this study
Asl-NG	(*95*)
Ubq-Spd-2-NG	*(55)*

Ubq-Msps-GFP	(*58*)
Ubq-Klp10A-GFP	Generated in this study
ncd-γ-tubulin-37c-GFP	(*117*)
UAS-CHC-GFP	(*118*)
Ubq-GFP-Cnn-ΔLZ	(*36*)
Ubq-GFP-Cnn-ΔCM2	(*36*)
(pSas-6)-mNG	(*95*) mNeonGreen expression driven by the Sas-6 promoter
NG-Sas-6	(*95*)
Ana2-NG	(*95*)
Spd-NG	(*101*)
NG-Cnn	(*101*)
Polo-TRAP-GFP	(*119*); appears to not be fully functional and is only viable as a heterozygote.
Ubq-RFP-Cnn	(*90*)
Ubq-mCherry-TACC	Generated in this study
Ubq-mCherry-TACC-S863L	Generated in this study
Jupiter-mCherry	(*120*)

Table ST2 *Drosophila* stocks used in specific experiments

Genotype	Purpose	Figure
GFP-Cnn	Comparing the characteristics of Cnn and TACC in cycling or colchicine-treated embryos	1A-B, E-F, 2A-B, S2
Ubq-GFP-TACC/+	• Comparing the characteristics of Cnn and TACC in cycling or colchicine-treated embryos	1A-B, D-F, 2A-B,

	• Comparing the properties of TACC in the presence or absence of endogenous Cnn	S1, S2, 3A
Ubq-GFP-TACC, Ubq-RFP-Cnn	Showing two scaffolds together in the same embryos	1C
cnn^{f04547} / cnn^{HK21} ; Ubq-GFP-TACC / +	• Comparing the properties of TACC in the presence or absence of endogenous Cnn<br>• Comparing the size of TACC in various genetic background in the absence of endogenous Cnn	1D, 3, S1
cnn^{f04547} / cnn^{HK21} ; Ubq-GFP-TACC / tacc^{stella592}	Comparing the size of TACC in various genetic background in the absence of endogenous Cnn	3
cnn^{f04547} / cnn^{HK21} ; Ubq-GFP-TACC / aur^{1}		
chc^{4}/+ ; cnn^{f04547} / cnn^{HK21} ; Ubq-GFP-TACC / +		
cnn^{f04547} / cnn^{HK21} ; Ubq-GFP-TACC / plp^{2172}		
cnn^{f04547} / cnn^{HK21} ; Ubq-GFP-TACC / Plk4^{Aa74}		
cnn^{f04547} / cnn^{HK21} ; Ubq-GFP-TACC / Spd-2^{z35711}		
Ubq-GFP-TACC-S863L/+	• Comparing the expression levels to Ubq-GFP-TACC	4A
cnn^{f04547} , RFP-Cnn / +; Ubq-GFP-TACC, tacc^{stella592} / +	Comparing the size of WT-TACC and non-phosphorylatable TACC-S863L mutants relative to the size of Cnn scaffold	4A-B
cnn^{f04547} , RFP-Cnn / +; Ubq-GFP-TACC-S863L, tacc^{stella592}/ +		
Ubq-NG-TACC, tacc^{59} / tacc^{74}	Comparing the recovery of WT-TACC and non-phosphorylatable TACC-S863L mutants in the absence of endogenous TACC	4C-D
Ubq-NG-TACC-S863L, tacc^{59} / tacc^{74}		
Ubq-Aurora A-GFP	Comparing the recruitment of Aurora A-GFP in embryos injected with different Spd-2 fused with mScarlet-I3	5C-D

Polo-TRAP-GFP	Comparing the recruitment of Polo-GFP in embryos injected with Spd-2-ΔPolo fused with mScarlet-I3	5E-F
Ubq-Aurora A-GFP / Ubq-Spd-2-RFP	Comparing the recovery of Spd-2 and Aurora A in the same embryos	6A-B
Ubq-Spd-2-SNAP / +; Spd-2-NG / Spd-2^{z35711}	Selective labelling of single molecules, Spd-2 single molecule behaviour	6C
Ubq-Spd-2-SNAP / +; Asl-NG / Spd-2^{z35711}	Measuring the distance between single Spd-2 molecules and the centriole labelled by Asl-NG	6D-E
Ubq-Spd-2-NG / +	Comparing the relative enrichment of various centrosomal proteins with or without Cnn scaffold	7A
cnn^{f04547} / cnn^{HK21} ; Ubq-Spd-2-NG / +		
Ubq-Aurora A-GFP / +		
cnn^{f04547} / cnn^{HK21} ; Ubq-Aurora A-GFP / +		
ncd-γ-tubulin-37c-GFP / +		
cnn^{f04547} / cnn^{HK21} ; ncd-γ-tubulin-37c-GFP / +		
Ubq-Msps-GFP / +		
cnn^{f04547} / cnn^{HK21} ; Ubq-Msps-GFP / +		
Ubq-Klp10A-GFP / +		
cnn^{f04547} / cnn^{HK21} ; Ubq-Klp10A-GFP / +		
V32a-Gal4 / + ; UAS-CHC-GFP / +		
cnn^{f04547}, V32a-Gal4 / cnn^{HK21} ; UAS-CHC-GFP / +		
cnn^{f04547}, Ubq-GFP-Cnn-ΔLZ / +	Showing Cnn-mutants that do not form scaffold can be concentrate to the TACC scaffold	7B
cnn^{f04547}, Ubq-GFP-Cnn-ΔLZ / cnn^{HK21}		

cnn^{f04547}, Ubq-GFP-Cnn-ΔCM2 / +		
cnn^{f04547}, Ubq-GFP-Cnn-ΔCM2 / cnn^{HK21}		
cnn^{HK21} / + ; (pSas-6)-mNG / Ubq-RFP-TACC	Comparing the concentration and diffusion coefficient of various centrosomal proteins inside and outside of TACC scaffold	7C-D, S5
cnn^{f04547} / cnn^{HK21} ; (pSas-6)-mNG / Ubq-RFP-TACC		
cnn^{HK21} / + ; NG-Sas-6/ Ubq-RFP-TACC		
cnn^{f04547} / cnn^{HK21} ; NG-Sas-6/ Ubq-RFP-TACC		
cnn^{f04547} , Ana2-NG / + ; Ubq-RFP-TACC / +		
cnn^{f04547} , Ana2-NG / cnn^{HK21} ; Ubq-RFP-TACC / +		
Spd-2-NG / Ubq-RFP-TACC		
NG-Cnn / + ; Ubq-RFP-TACC / +		
cnn^{f04547} , RFP-Cnn / +	Comparing the localisation of RFP-Cnn, mCherry-TACC or mCherry-Jupiter on synthetic beads coated with α-GFP nanobody in cycling embryos co-injected various GFP-fused centrosomal proteins	8C-D, S6
Ubq-mCherry-TACC		
Jupiter-mCherry		
cnn^{f04547} , RFP-Cnn / Polo-TRAP-GFP	Comparing the localisation of RFP-Cnn, mCherry-TACC or mCherry-Jupiter co-expressed with Polo-GFP or Aurora-GFP on synthetic beads coated with α-ALFA nanobody in cycling embryos co-injected various Spd-2 mutants fused with ALFA-tag	9
Ubq-mCherry-TACC / Aurora A-GFP		
Jupiter-mCherry / Polo-TRAP-GFP		
Jupiter-mCherry / Aurora A-GFP		
Spd-2-RFP / GFP-TACC	Observing the nucleation and dissipation of TACC scaffold on centrioles	S4

Table ST3 Primers used in this study

Index	Primer	Purpose
s1	*5′-ATG CAA AAC TAA ACG GTG TCA TTG AGG CAT ATG AGA AGG CAA TTG CAG AGC TCA TTA GTG AGA AGG AGC*	Amplifying WT-TACC cDNA and adding attB sites on both ends of the cDNA, which allowed the cDNA to be flipped into a pDonor plasmid
s2	*5′-TTG TTT TGT CCT TTT CTG TAA TAC GCT TGA TCA GAG CCT CCT CGC GCA GCT CTC CCT CCT TGA GC*	
s3	*5′-TGC TGC TGT TGA AGT TTG ATC CCC TCC TTG*	Mutating Aurora A phosphorylation site Serine 863 on WT-TACC to a Leucine
s4	*5′-GAC TGC GGT CAA TAG GCA CAC TAT TGC TG*	
s5	*5′-CCG GCG GCA GCT AAG CGG CCG CGG ATC TGG TTAC*	Linearising pRNA destination vector backbone with overlapping regions with mScarlet-I3
s6	*5′- GGT GCT ATC CAT GCT AGA TCG AAC CAC TTT GTA CAA GAA AGC TG*	
s7	*5′-TGG CGG CGG AAG GCC AGG ATA CGG CCA TGC CGA CGG CCA CGT TG*	Deleting the region on Spd-2 that contains ABD1
s8	*5′-ATC CTG GCC TTC CGC CGC CAA C*	
s9	*5′-TGG CCG GAG ATC CAG ACT TTA GCC CGA ATC GTT CGC GAA C*	Deleting the region on Spd-2 that contains ABD2
s10	*5′-AAA GTC TGG ATC TCC GGC CAA G*	
s11	*5′-AGA AGA ACT GCG TCG TCG TCT GAC CGA ATA AAT GGT GAG CAA GGG CGA GG*	Replacing GFP on various versions of pRNA-Spd-2-GFP vectors with ALFA-tag
s12	*5′-ACG ACG ACG CAG TTC TTC TTC CAG ACG AGA GCT AGA TCG AAC CAC TTT G*	
s13	*5′-* ATC CAG CAC AGT GGC GGC CGC TCG AGG CTA GCA TGG ACA AAG ACT GCG AAA TGA AGC GCA CC	Cloning codon-optimised SNAP-tag into pUbq destination vector
s14	*5′-* CTA GTT CTA GAG CGG CCG CCA CCG CGG TTA ACC CAG CCC AGG CTT GCC CAG TCT GT	
s15	*5′-TGG GTC GCG GAT CCG ATC AAG TTC AGT TAC AAG AGA GTG GGG G*	Amplifying anti-ALFA nanobody

s16	*5′- GGA TAG TCG AAT TCA CTA GTG CTG CTC ACA GTC ACT TGGG TG*	
s17	*5′-ACT AGT GAA TTC GAC TAT CCA TAT G*	Replacing anti-GFP nanobody cDNA on pET24a-VHH-std vector with anti-ALFA nanobody cDNA
s18	*5′-TTG ATC GGA TCC GCG ACC CAT TTG C*	

Table ST4 Plasmids used in this study

Index	Plasmid	Purpose	Source
p1	pDonor-TACC-WT	Donating WT-TACC to various pDestination vectors	Generated in Raff Lab
p2	pDonor-TACC-S863L	Donating TACC-S863L to various pDestination vectors	Generated in Raff Lab
p3	pUbq-NG-NT destination	Adding NG to the N-terminus of donor cDNA for transgenic fly generation	(*56*)
p4	pUbq-mCherry-NT destination	Adding mCherry to the N-terminus of donor cDNA for transgenic fly generation	Generated in Raff Lab
p5	pUbq-NG-TACC-WT	Generating transgenic fly line expressing N-terminally NG-fused WT-TACC	Generated in Raff Lab
p6	pUbq-NG-TACC-S863L	Generating transgenic fly line expressing N-terminally NG-fused TACC-S863L	Generated in Raff Lab
p7	pUbq-mCherry-TACC-WT	Generating transgenic fly line expressing N-terminally mCherry-fused WT-TACC	Generated in Raff Lab
p8	pUbq-GFP-CT destination	Adding GFP to the C-terminus of donor cDNA for transgenic fly generation	(*121*)
p9	pUbq-Klp10A-GFP	Generating transgenic fly line expressing C-terminally GFP-fused Klp10A	Generated in Raff Lab
p10	pRNA-GFP-CT destination	Adding GFP to the C-terminus of donor cDNA for mRNA synthesis	(*122*)
p11	pDonor-Spd-2-WT	Donating Spd-2-WT to various pDestination vectors	(*28*)
p12	pRNA-Spd-2-GFP	Synthesizing mRNA encoding C-terminally GFP-fused WT-Spd-2	Generated in Raff Lab
p13	pRNA-GFP-NT destination	Adding GFP to the N-terminus of donor cDNA for mRNA synthesis	(*33*)

p14	pDonor-Cnn-WT	Donating Cnn-WT to various pDestination vectors	(*33*)
p15	pRNA-GFP-Cnn	Synthesizing mRNA encoding N-terminally GFP-fused WT-Cnn	Generated in Raff Lab
p16	pRNA-GFP-TACC	Synthesizing mRNA encoding N-terminally GFP-fused WT-TACC	Generated in Raff Lab
p17	pDRESS_mTurquoise2_spatial-linker-P2A_mScarlet-I3	mScarlet-I3 containing plasmid	Addgene #189755
p18	pRNA-mScarlet-I3 Destination	Adding mScarlet-I3 to the C-terminus of donor cDNA for mRNA synthesis	Generated in Raff Lab
p19	pDonor-Spd-2ΔPolo	Donating Spd-2-ΔPolo to various pDestination vectors	(*28*)
p20	pRNA-Spd-2ΔPolo-mScarlet-I3	Synthesizing mRNA encoding C-terminally mScarlet-I3-fused Spd-2-ΔPolo	Generated in Raff Lab
p21	pRNA-Spd-2ΔABD1-mScarlet-I3	Synthesizing mRNA encoding C-terminally mScarlet-I3-fused Spd-2-ΔABD1	Generated in Raff Lab
p22	pRNA-Spd-2ΔABD2-mScarlet-I3	Synthesizing mRNA encoding C-terminally mScarlet-I3-fused Spd-2-ΔABD2	Generated in Raff Lab
p23	pRNA-Spd-2ΔABD1-ALFA	Synthesizing mRNA encoding C-terminally ALFA-fused Spd-2-ΔABD1	Generated in Raff Lab
p24	pRNA-Spd-2ΔABD2-ALFA	Synthesizing mRNA encoding C-terminally ALFA-fused Spd-2-ΔABD2	Generated in Raff Lab
p25	pRNA-Spd-2ΔPolo-ΔABD2-ALFA	Synthesizing mRNA encoding C-terminally ALFA-fused Spd-2-ΔPolo-ΔABD2	Generated in Raff Lab
p26	pUbq-SNAP Destination	Adding SNAP to the C-terminus of donor cDNA for transgenic fly generation	Generated in Raff Lab
p27	pUbq-Spd-2-SNAP	Generating transgenic fly line expressing C-terminally SNAP-fused Spd-2	Generated in Raff Lab
p28	cfSGFP2-anti-AlfaTag nanobody	anti-ALFA nanobody containing plasmid	Addgene #171818

p29	pET24a-VHH-std	Expressing anti-GFP nanobody	Addgene #109417
p30	pET24a-anti-ALFA-std	Expressing anti-ALFA nanobody	Generated in Raff Lab
p31	pET-21d-myc-BirA	Expressing BirA with anti-GFP nanobody or anti-ALFA nanobody for nanobody biotinylation	Addgene #109424

REFERENCES AND NOTES

1. M. Bornens, Centrosome organization and functions. *Curr. Opin. Struct. Biol.* **66**, 199–206 (2021).

2. A. Vasquez-Limeta, J. Loncarek, Human centrosome organization and function in interphase and mitosis. *Semin. Cell Dev. Biol.* **117**, 30–41 (2021).

3. S. Gomes Pereira, M. A. Dias Louro, M. Bettencourt-Dias, Biophysical and quantitative principles of centrosome biogenesis and structure. *Annu. Rev. Cell Dev. Biol.* **37**, 43–63 (2021).

4. P. T. Conduit, A. Wainman, J. W. Raff, Centrosome function and assembly in animal cells. *Nat. Rev. Mol. Cell Biol.* **16**, 611–624 (2015).

5. J. M. da Conceiçao Alves-Cruzeiro, R. Nogales-Cadenas, A. D. Pascual-Montano, CentrosomeDB: A new generation of the centrosomal proteins database for *Human* and *Drosophila melanogaster*. *Nucleic Acids Res.* **42**, D430–D436 (2014).

6. Z. Huang, L. Ma, Y. Wang, Z. Pan, J. Ren, Z. Liu, Y. Xue, MiCroKiTS 4.0: A database of midbody, centrosome, kinetochore, telomere and spindle. *Nucleic Acids Res.* **43**, D328–D334 (2015).

7. O. Goundiam, R. Basto, Centrosomes in disease: How the same music can sound so different? *Curr. Opin. Struct. Biol.* **66**, 74–82 (2021).

8. S. A. Godinho, D. Pellman, Causes and consequences of centrosome abnormalities in cancer. *Philos. Trans. R. Soc. B Biol. Sci.* **369**, 20130467 (2014).

9. M. Bettencourt-Dias, F. Hildebrandt, D. Pellman, G. Woods, S. A. Godinho, Centrosomes and cilia in human disease. *Trends Genet.* **27**, 307–315 (2011).

10. E. A. Nigg, J. W. Raff, Centrioles, centrosomes, and cilia in health and disease. *Cell* **139**, 663–678 (2009).

11. P. Meraldi, Centrosomes in spindle organization and chromosome segregation: A mechanistic view. *Chromosome Res.* **24**, 19–34 (2016).

12. R. E. Palazzo, J. M. Vogel, B. J. Schnackenberg, D. R. Hull, X. Wu, Centrosome maturation. *Curr. Top. Dev. Biol.* **49**, 449–470 (2000).

13. S. J. Enos, M. Dressler, B. F. Gomes, A. A. Hyman, J. B. Woodruff, Phosphatase PP2A and microtubule-mediated pulling forces disassemble centrosomes during mitotic exit. *Biol. Open* **7**, bio029777 (2018).

14. J. Magescas, J. C. Zonka, J. L. Feldman, A two-step mechanism for the inactivation of microtubule organizing center function at the centrosome. *eLife* **8**, e47867 (2019).

15. M. Mittasch, V. M. Tran, M. U. Rios, A. W. Fritsch, S. J. Enos, B. Ferreira Gomes, A. Bond, M. Kreysing, J. B. Woodruff, Regulated changes in material properties underlie centrosome disassembly during mitotic exit. *J. Cell Biol.* **219** (2020).

16. M. J. Rale, R. S. Kadzik, S. Petry, Phase transitioning the centrosome into a microtubule nucleator. *Biochemistry* **57**, 30–37 (2018).

17. J. B. Woodruff, Assembly of mitotic structures through phase separation. *J. Mol. Biol.* **430**, 4762–4772 (2018).

18. J. W. Raff, Phase separation and the centrosome: A fait accompli? *Trends Cell Biol.* **29**, 612–622 (2019).

19. K. S. Lee, J.-E. Park, J. I. Ahn, Y. Zeng, Constructing PCM with architecturally distinct higher-order assemblies. *Curr. Opin. Struct. Biol.* **66**, 66–73 (2021).

20. J. B. Woodruff, The material state of centrosomes: Lattice, liquid, or gel? *Curr. Opin. Struct. Biol.* **66**, 139–147 (2021).

21. Y. Shin, C. P. Brangwynne, Liquid phase condensation in cell physiology and disease. *Science* **357**, eaaf4382 (2017).

22. S. Alberti, A. A. Hyman, Biomolecular condensates at the nexus of cellular stress, protein aggregation disease and ageing. *Nat. Rev. Mol. Cell Biol.* **22**, 196–213 (2021).

23. K. F. O'Connell, K. N. Maxwell, J. G. White, The *spd-2* gene is required for polarization of the anteroposterior axis and formation of the sperm asters in the *Caenorhabditis elegans* zygote. *Dev. Biol.* **222**, 55–70 (2000).

24. C. A. Kemp, K. R. Kopish, P. Zipperlen, J. Ahringer, K. F. O'Connell, Centrosome maturation and duplication in C. elegans require the coiled-coil protein SPD-2. *Dev. Cell* **6**, 511–523 (2004).

25. C. I. Dix, J. W. Raff, Drosophila Spd-2 recruits PCM to the sperm centriole, but is dispensable for centriole duplication. *Curr. Biol.* **17**, 1759–1764 (2007).

26. M. G. Giansanti, E. Bucciarelli, S. Bonaccorsi, M. Gatti, *Drosophila* SPD-2 is an essential centriole component required for PCM recruitment and astral-microtubule nucleation. *Curr. Biol.* **18**, 303–309 (2008).

27. M. Decker, S. Jaensch, A. Pozniakovsky, A. Zinke, K. F. O'Connell, W. Zachariae, E. Myers, A. A. Hyman, Limiting amounts of centrosome material set centrosome size in *C. elegans* embryos. *Curr. Biol.* **21**, 1259–1267 (2011).

28. I. Alvarez Rodrigo, T. L. Steinacker, S. Saurya, P. T. Conduit, J. Baumbach, Z. A. Novak, M. G. Aydogan, A. Wainman, J. W. Raff, Evidence that a positive feedback loop drives centrosome maturation in fly embryos. *eLife* **8**, e50130 (2019).

29. I. Alvarez-Rodrigo, A. Wainman, S. Saurya, J. W. Raff, Ana1 helps recruit Polo to centrioles to promote mitotic PCM assembly and centriole elongation. *J. Cell Sci.* **134**, jcs258987 (2021).

30. J. B. Woodruff, O. Wueseke, V. Viscardi, J. Mahamid, S. D. Ochoa, J. Bunkenborg, P. O. Widlund, A. Pozniakovsky, E. Zanin, S. Bahmanyar, A. Zinke, S. H. Hong, M. Decker, W. Baumeister, J. S. Andersen, K. Oegema, A. A. Hyman, Regulated assembly of a supramolecular centrosome scaffold in vitro. *Science* **348**, 808–812 (2015).

31. G. Cabral, T. Laos, J. Dumont, A. Dammermann, Differential requirements for centrioles in mitotic centrosome growth and maintenance. *Dev. Cell* **50**, 355–366.e6 (2019).

32. M. Ohta, Z. Zhao, D. Wu, S. Wang, J. L. Harrison, J. S. Gómez-Cavazos, A. Desai, K. F. Oegema, Polo-like kinase 1 independently controls microtubule-nucleating capacity and size of the centrosome. *J. Cell Biol.* **220**, e202009083 (2021).

33. P. T. Conduit, Z. Feng, J. H. Richens, J. Baumbach, A. Wainman, S. D. Bakshi, J. Dobbelaere, S. Johnson, S. M. Lea, J. W. Raff, The centrosome-specific phosphorylation of Cnn by Polo/Plk1 drives Cnn scaffold assembly and centrosome maturation. *Dev. Cell* **28**, 659–669 (2014).

34. P. T. Conduit, J. H. Richens, A. Wainman, J. Holder, C. C. Vicente, M. B. Pratt, C. I. Dix, Z. A. Novak, I. M. Dobbie, L. Schermelleh, J. W. Raff, A molecular mechanism of mitotic centrosome assembly in Drosophila. *eLife* **3**, e03399 (2014).

35. J. B. Woodruff, B. Ferreira Gomes, P. O. Widlund, J. Mahamid, A. Honigmann, A. A. Hyman, The centrosome is a selective condensate that nucleates microtubules by concentrating tubulin. *Cell* **169**, 1066–1077.e10 (2017).

36. Z. Feng, A. Caballe, A. Wainman, S. Johnson, A. F. M. Haensele, M. A. Cottee, P. T. Conduit, S. M. Lea, J. W. Raff, Structural basis for mitotic centrosome assembly in flies. *Cell* **169**, 1078–1089.e13 (2017).

37. M. Nakajo, H. Kano, K. Tsuyama, N. Haruta, A. Sugimoto, Centrosome maturation requires phosphorylation-mediated sequential domain interactions of SPD-5. *J. Cell Sci.* **135**, jcs259025 (2022).

38. F. Zhu, S. Lawo, A. Bird, D. Pinchev, A. Ralph, C. Richter, T. Müller-Reichert, R. Kittler, A. A. Hyman, L. Pelletier, The mammalian SPD-2 ortholog Cep192 regulates centrosome biogenesis. *Curr. Biol.* **18**, 136–141 (2008).

39. M. A. Gomez-Ferreria, U. Rath, D. W. Buster, S. K. Chanda, J. S. Caldwell, D. R. Rines, D. J. Sharp, Human Cep192 is required for mitotic centrosome and spindle assembly. *Curr. Biol.* **17**, 1960–1966 (2007).

40. V. Joukov, A. De Nicolo, A. Rodriguez, J. C. Walter, D. M. Livingston, Centrosomal protein of 192 kDa (Cep192) promotes centrosome-driven spindle assembly by engaging in organelle-specific Aurora A activation. *Proc. Natl. Acad. Sci. U.S.A.* **107**, 21022–21027 (2010).

41. T. Chinen, K. Yamazaki, K. Hashimoto, K. Fujii, K. Watanabe, Y. Takeda, S. Yamamoto, Y. Nozaki, Y. Tsuchiya, D. Takao, D. Kitagawa, Centriole and PCM cooperatively recruit CEP192 to spindle poles to promote bipolar spindle assembly. *J. Cell Biol.* **220**, e202006085 (2021).

42. H. A. Lane, E. A. Nigg, Antibody microinjection reveals an essential role for human polo-like kinase 1 (Plk1) in the functional maturation of mitotic centrosomes. *J. Cell Biol.* **135**, 1701–1713 (1996).

43. L. Haren, T. Stearns, J. Lüders, Plk1-dependent recruitment of gamma-tubulin complexes to mitotic centrosomes involves multiple PCM components. *PLOS ONE* **4**, e5976 (2009).

44. K. Lee, K. Rhee, PLK1 phosphorylation of pericentrin initiates centrosome maturation at the onset of mitosis. *J. Cell Biol.* **195**, 1093–1101 (2011).

45. V. Joukov, J. C. Walter, A. De Nicolo, The Cep192-organized aurora A-Plk1 cascade is essential for centrosome cycle and bipolar spindle assembly. *Mol. Cell* **55**, 578–591 (2014).

46. L. Meng, J.-E. Park, T.-S. Kim, E. H. Lee, S.-Y. Park, M. Zhou, J. K. Bang, K. S. Lee, Bimodal interaction of mammalian polo-like kinase 1 and a centrosomal scaffold, Cep192, in the regulation of bipolar spindle formation. *Mol. Cell. Biol.* **35**, 2626–2640 (2015).

47. K.-W. Fong, Y.-K. Choi, J. B. Rattner, R. Z. Qi, CDK5RAP2 is a pericentriolar protein that functions in centrosomal attachment of the gamma-tubulin ring complex. *Mol. Biol. Cell* **19**, 115–125 (2008).

48. Y.-K. Choi, P. Liu, S. K. Sze, C. Dai, R. Z. Qi, CDK5RAP2 stimulates microtubule nucleation by the gamma-tubulin ring complex. *J. Cell Biol.* **191**, 1089–1095 (2010).

49. S. B. Lizarraga, S. P. Margossian, M. H. Harris, D. R. Campagna, A.-P. Han, S. Blevins, R. Mudbhary, J. E. Barker, C. A. Walsh, M. D. Fleming, Cdk5rap2 regulates centrosome function and chromosome segregation in neuronal progenitors. *Development* **137**, 1907–1917 (2010).

50. A. R. Barr, J. V. Kilmartin, F. Gergely, CDK5RAP2 functions in centrosome to spindle pole attachment and DNA damage response. *J. Cell Biol.* **189**, 23–39 (2010).

51. P. Tátrai, F. Gergely, Centrosome function is critical during terminal erythroid differentiation. *EMBO J.* **41**, e108739 (2022).

52. C. So, K. B. Seres, A. M. Steyer, E. Mönnich, D. Clift, A. Pejkovska, W. Möbius, M. Schuh, A liquid-like spindle domain promotes acentrosomal spindle assembly in mammalian oocytes. *Science* **364**, eaat9557 (2019).

53. F. Gergely, C. Karlsson, I. Still, J. Cowell, J. Kilmartin, J. W. Raff, The TACC domain identifies a family of centrosomal proteins that can interact with microtubules. *Proc. Natl. Acad. Sci. U.S.A.* **97**, 14352–14357 (2000).

54. F. Gergely, D. Kidd, K. Jeffers, J. G. Wakefield, J. W. Raff, D-TACC: A novel centrosomal protein required for normal spindle function in the early *Drosophila* embryo. *EMBO J.* **19**, 241–252 (2000).

55. S.-S. Wong, Z. M. Wilmott, S. Saurya, I. Alvarez-Rodrigo, F. Y. Zhou, K.-Y. Chau, A. Goriely, J. W. Raff, Centrioles generate a local pulse of Polo/PLK1 activity to initiate mitotic centrosome assembly. *EMBO J.* **41**, e110891 (2022).

56. S.-S. Wong, A. Wainman, S. Saurya, J. W. Raff, Regulation of centrosome size by the cell-cycle oscillator in *Drosophila* embryos. *EMBO J.* **43**, 414–436 (2024).

57. T. L. Megraw, S. Kilaru, F. R. Turner, T. C. Kaufman, The centrosome is a dynamic structure that ejects PCM flares. *J. Cell Sci.* **115**, 4707–4718 (2002).

58. M. J. Lee, F. Gergely, K. Jeffers, S. Y. Peak-Chew, J. W. Raff, Msps/XMAP215 interacts with the centrosomal protein D-TACC to regulate microtubule behaviour. *Nat. Cell Biol.* **3**, 643–649 (2001).

59. X. Wang, C. Baumann, R. De La Fuente, M. M. Viveiros, Loss of acentriolar MTOCs disrupts spindle pole Aurora A and assembly of the liquid-like meiotic spindle domain in oocytes. *J. Cell Sci.* **134**, jcs256297 (2021).

60. C. S. Blengini, P. Ibrahimian, M. Vaskovicova, D. Drutovic, P. Solc, K. Schindler, Aurora kinase A is essential for meiosis in mouse oocytes. *PLOS Genet.* **17**, e1009327 (2021).

61. J. H. Richens, T. P. Barros, E. P. Lucas, N. Peel, D. M. S. Pinto, A. Wainman, J. W. Raff, The *Drosophila* Pericentrin-like-protein (PLP) cooperates with Cnn to maintain the integrity of the outer PCM. *Biol. Open* **4**, 1052–1061 (2015).

62. D. A. Lerit, H. A. Jordan, J. S. Poulton, C. J. Fagerstrom, B. J. Galletta, M. Peifer, N. M. Rusan, Interphase centrosome organization by the PLP-Cnn scaffold is required for centrosome function. *J. Cell Biol.* **210**, 79–97 (2015).

63. M. Bettencourt-Dias, A. Rodrigues-Martins, L. Carpenter, M. Riparbelli, L. Lehmann, M. K. Gatt, N. Carmo, F. Balloux, G. Callaini, D. M. Glover, SAK/PLK4 is required for centriole duplication and flagella development. *Curr. Biol.* **15**, 2199–2207 (2005).

64. M. G. Aydogan, T. L. Steinacker, M. Mofatteh, Z. M. Wilmott, F. Y. Zhou, L. Gartenmann, A. Wainman, S. Saurya, Z. A. Novak, S.-S. Wong, A. Goriely, M. A. Boemo, J. W. Raff, An autonomous oscillation times and executes centriole biogenesis. *Cell* **181**, 1566–1581.e27 (2020).

65. T. P. Barros, K. Kinoshita, A. A. Hyman, J. W. Raff, Aurora A activates D-TACC-Msps complexes exclusively at centrosomes to stabilize centrosomal microtubules. *J. Cell Biol.* **170**, 1039–1046 (2005).

66. K. Kinoshita, T. L. Noetzel, L. Pelletier, K. Mechtler, D. N. Drechsel, A. Schwager, M. Lee, J. W. Raff, A. A. Hyman, Aurora A phosphorylation of TACC3/maskin is required for centrosome-dependent microtubule assembly in mitosis. *J. Cell Biol.* **170**, 1047–1055 (2005).

67. C.-H. Lin, C.-K. Hu, H.-M. Shih, Clathrin heavy chain mediates TACC3 targeting to mitotic spindles to ensure spindle stability. *J. Cell Biol.* **189**, 1097–1105 (2010).

68. D. G. Booth, F. E. Hood, I. A. Prior, S. J. Royle, A TACC3/ch-TOG/clathrin complex stabilises kinetochore fibres by inter-microtubule bridging. *EMBO J.* **30**, 906–919 (2011).

69. F. E. Hood, S. J. Williams, S. G. Burgess, M. W. Richards, D. Roth, A. Straube, M. Pfuhl, R. Bayliss, S. J. Royle, Coordination of adjacent domains mediates TACC3–ch-TOG–clathrin assembly and mitotic spindle binding. *J. Cell Biol.* **202**, 463–478 (2013).

70. J.-G. Park, H. Jeon, S. Shin, C. Song, H. Lee, N.-K. Kim, E. E. Kim, K. Y. Hwang, B.-J. Lee, I.-G. Lee, Structural basis for CEP192-mediated regulation of centrosomal AURKA. *Sci. Adv.* **9**, eadf8582 (2023).

71. V. Joukov, A. De Nicolo, Aurora-PLK1 cascades as key signaling modules in the regulation of mitosis. *Sci. Signal.* **11**, eaar4195 (2018).

72. R. Bayliss, T. Sardon, I. Vernos, E. Conti, Structural basis of Aurora-A activation by TPX2 at the mitotic spindle. *Mol. Cell* **12**, 851–862 (2003).

73. J. Holder, J. A. Miles, M. Batchelor, H. Popple, M. Walko, W. Yeung, N. Kannan, A. J. Wilson, R. Bayliss, F. Gergely, CEP192 localises mitotic Aurora-A activity by priming its interaction with TPX2. *EMBO J.* **43**, 5381–5420 (2024).

74. T. W. J. Gadella, L. van Weeren, J. Stouthamer, M. A. Hink, A. H. G. Wolters, B. N. G. Giepmans, S. Aumonier, J. Dupuy, A. Royant, mScarlet3: A brilliant and fast-maturing red fluorescent protein. *Nat. Methods* **20**, 541–545 (2023).

75. Z. A. Novak, A. Wainman, L. Gartenmann, J. W. Raff, Cdk1 phosphorylates *Drosophila* Sas-4 to recruit Polo to daughter centrioles and convert them to centrosomes. *Dev. Cell* **37**, 545–557 (2016).

76. A. Keppler, S. Gendreizig, T. Gronemeyer, H. Pick, H. Vogel, K. Johnsson, A general method for the covalent labeling of fusion proteins with small molecules in vivo. *Nat. Biotechnol.* **21**, 86–89 (2003).

77. J. B. Grimm, B. P. English, J. Chen, J. P. Slaughter, Z. Zhang, A. Revyakin, R. Patel, J. J. Macklin, D. Normanno, R. H. Singer, T. Lionnet, L. D. Lavis, A general method to improve fluorophores for live-cell and single-molecule microscopy. *Nat. Methods* **12**, 244–250 (2015).

78. Z. A. Novak, P. T. Conduit, A. Wainman, J. W. Raff, Asterless licenses daughter centrioles to duplicate for the first time in Drosophila embryos. *Curr. Biol.* **24**, 1276–1282 (2014).

79. A. Dammermann, T. Müller-Reichert, L. Pelletier, B. Habermann, A. Desai, K. Oegema, Centriole assembly requires both centriolar and pericentriolar material proteins. *Dev. Cell* **7**, 815–829 (2004).

80. C. Arquint, E. A. Nigg, The PLK4-STIL-SAS-6 module at the core of centriole duplication. *Biochem. Soc. Trans.* **44**, 1253–1263 (2016).

81. P. Gönczy, G. N. Hatzopoulos, Centriole assembly at a glance. *J. Cell Sci.* **132**, jcs228833 (2019).

82. H. Götzke, M. Kilisch, M. Martínez-Carranza, S. Sograte-Idrissi, A. Rajavel, T. Schlichthaerle, N. Engels, R. Jungmann, P. Stenmark, F. Opazo, S. Frey, The ALFA-tag

is a highly versatile tool for nanobody-based bioscience applications. *Nat. Commun.* **10**, 4403 (2019).

83. I. A. Asteriti, F. De Mattia, G. Guarguaglini, Cross-talk between AURKA and Plk1 in mitotic entry and spindle assembly. *Front. Oncol.* **5**, 283 (2015).

84. N. Le Bot, M.-C. Tsai, R. K. Andrews, J. Ahringer, TAC-1, a regulator of microtubule length in the *C. elegans* embryo. *Curr. Biol.* **13**, 1499–1505 (2003).

85. M. Hedtfeld, A. Dammers, C. Koerner, A. Musacchio, A validation strategy to assess the role of phase separation as a determinant of macromolecular localization. *Mol. Cell* **84**, 1783–1801.e7 (2024).

86. S. G. Burgess, M. Mukherjee, S. Sabir, N. Joseph, C. Gutiérrez-Caballero, M. W. Richards, N. Huguenin-Dezot, J. W. Chin, E. J. Kennedy, M. Pfuhl, S. J. Royle, F. Gergely, R. Bayliss, Mitotic spindle association of TACC3 requires Aurora-A-dependent stabilization of a cryptic α-helix. *EMBO J.* **37**, e97902 (2018).

87. A. Musacchio, On the role of phase separation in the biogenesis of membraneless compartments. *EMBO J.* **41**, e109952 (2022).

88. J. Zhang, T. L. Megraw, Proper recruitment of gamma-tubulin and D-TACC/Msps to embryonic Drosophila centrosomes requires Centrosomin Motif 1. *Mol. Biol. Cell* **18**, 4037–4049 (2007).

89. C. A. Tovey, C. Tsuji, A. Egerton, F. Bernard, A. Guichet, M. de la Roche, P. T. Conduit, Autoinhibition of Cnn binding to γ-TuRCs prevents ectopic microtubule nucleation and cell division defects. *J. Cell Biol.* **220**, e202010020 (2021).

90. E. P. Lucas, J. W. Raff, Maintaining the proper connection between the centrioles and the pericentriolar matrix requires Drosophila centrosomin. *J. Cell Biol.* **178**, 725–732 (2007).

91. S. G. Burgess, I. Peset, N. Joseph, T. Cavazza, I. Vernos, M. Pfuhl, F. Gergely, R. Bayliss, Aurora-A-dependent control of TACC3 influences the rate of mitotic spindle assembly. *PLOS Genet.* **11**, e1005345 (2015).

92. E. B. Roberts, *Drosophila A Practical Approach* (Oxford Univ. Press, 1998); http://scholar.google.com/scholar?q=related:emfVbOEMAZ8J:scholar.google.com/&hl=en&num=20&as_sdt=0,5.

93. S. J. Radford, A. M. Harrison, K. S. McKim, Microtubule-depolymerizing kinesin KLP10A restricts the length of the acentrosomal meiotic spindle in Drosophila females. *Genetics* **192**, 431–440 (2012).

94. D. P. Buser, K. D. Schleicher, C. Prescianotto-Baschong, M. Spiess, A versatile nanobody-based toolkit to analyze retrograde transport from the cell surface. *Proc. Natl. Acad. Sci. U.S.A.* **115**, E6227–E6236 (2018).

95. T. L. Steinacker, S.-S. Wong, Z. A. Novak, S. Saurya, L. Gartenmann, E. J. H. van Houtum, J. R. Sayers, B. C. Lagerholm, J. W. Raff, Centriole growth is limited by the Cdk/Cyclin-dependent phosphorylation of Ana2/STIL. *J. Cell Biol.* **221**, e202205058 (2022).

96. J. C. Crocker, D. G. Grier, Methods of digital video microscopy for colloidal studies. *J. Colloid Interface Sci.* **179**, 298–310 (1996).

97. D. Allan, T. Caswell, N. Keim, C. van der Wel, trackpy: Trackpy v0.3.2, version v0.3.2 (Zenodo, 2016); https://doi.org/10.5281/zenodo.60550.

98. N. Otsu, A threshold selection method from gray-level histograms. *IEEE Trans. Syst. Man Cybern.* **9**, 62–66 (1979).

99. P. Soille, Background notions, in *Morphological Image Analysis: Principles and Applications*, P. Soille, Ed. (Springer, 2004); https://doi.org/10.1007/978-3-662-05088-0_2)10.1007/978-3-662-05088-0_2, pp. 15–62.

100. J. Cartucho, R. Ventura, M. Veloso, Robust object recognition through symbiotic deep learning in mobile robots, in *2018 IEEE/RSJ International Conference on Intelligent Robots and Systems (IROS)* (IEEE, 2018), pp. 2336–2341; https://ieeexplore.ieee.org/document/8594067.

101. S.-S. Wong, Z. M. Wilmott, S. Saurya, F. Y. Zhou, K.-Y. Chau, A. Goriely, J. W. Raff, Mother centrioles generate a local pulse of Polo/PLK1 activity to initiate mitotic centrosome assembly. bioRxiv 465695 [Preprint] (2021). https://doi.org/10.1101/2021.10.26.465695.

102. J.-Y. Tinevez, N. Perry, J. Schindelin, G. M. Hoopes, G. D. Reynolds, E. Laplantine, S. Y. Bednarek, S. L. Shorte, K. W. Eliceiri, TrackMate: An open and extensible platform for single-particle tracking. *Methods* **115**, 80–90 (2017).

103. R. M. Dickson, A. B. Cubitt, R. Y. Tsien, W. E. Moerner, On/off blinking and switching behaviour of single molecules of green fluorescent protein. *Nature* **388**, 355–358 (1997).

104. M. Mirdita, K. Schütze, Y. Moriwaki, L. Heo, S. Ovchinnikov, M. Steinegger, ColabFold: Making protein folding accessible to all. *Nat. Methods* **19**, 679–682 (2022).

105. E. F. Pettersen, T. D. Goddard, C. C. Huang, G. S. Couch, D. M. Greenblatt, E. C. Meng, T. E. Ferrin, UCSF Chimera—A visualization system for exploratory research and analysis. *J. Comput. Chem.* **25**, 1605–1612 (2004).

106. N. R. Stevens, J. Dobbelaere, A. Wainman, F. Gergely, J. W. Raff, Ana3 is a conserved protein required for the structural integrity of centrioles and basal bodies. *J. Cell Biol.* **187**, 355–363 (2009).

107. J. W. Raff, R. Kellum, B. Alberts, The Drosophila GAGA transcription factor is associated with specific regions of heterochromatin throughout the cell cycle. *EMBO J.* **13**, 5977–5983 (1994).

108. M. Hartley, G. J. Kleywegt, A. Patwardhan, U. Sarkans, J. R. Swedlow, A. Brazma, The BioImage Archive - building a home for life-sciences microscopy data. *J. Mol. Biol.* **434**, 167505 (2022).

109. T. L. Megraw, K. Li, L. R. Kao, T. C. Kaufman, The centrosomin protein is required for centrosome assembly and function during cleavage in Drosophila. *Development* **126**, 2829–2839 (1999).

110. D. Vaizel-Ohayon, E. D. Schejter, Mutations in centrosomin reveal requirements for centrosomal function during early Drosophila embryogenesis. *Curr. Biol.* **9**, 889–898 (1999).

111. C. A. Tovey, C. Tsuji, A. Egerton, F. Bernard, A. Guichet, M. De, L. Roche, P. T. Conduit, Phospho-regulated auto-inhibition of Cnn controls microtubule nucleation during cell division. bioRxiv 326587 [Preprint] (2020). https://doi.org/10.1101/2020.10.05.326587.

112. D. M. Glover, M. H. Leibowitz, D. A. McLean, H. Parry, Mutations in *aurora* prevent centrosome separation leading to the formation of monopolar spindles. *Cell* **81**, 95–105 (1995).

113. A. C. Spradling, D. Stern, A. Beaton, E. J. Rhem, T. Laverty, N. Mozden, S. Misra, G. Rubin, The Berkeley Drosophila Genome Project gene disruption project: Single P-element insertions mutating 25% of vital Drosophila genes. *Genetics* **153**, 135–177 (1999).

114. M. G. Aydogan, A. Wainman, S. Saurya, T. L. Steinacker, A. Caballe, Z. A. Novak, J. Baumbach, N. Muschalik, J. W. Raff, A homeostatic clock sets daughter centriole size in flies. *J. Cell Biol.* **217**, 1233–1248 (2018).

115. P. T. Conduit, K. Brunk, J. Dobbelaere, C. I. Dix, E. P. Lucas, J. W. Raff, Centrioles regulate centrosome size by controlling the rate of Cnn incorporation into the PCM. *Curr. Biol.* **20**, 2178–2186 (2010).

116. Q. Tang, M. Rui, S. Bu, Y. Wang, L. Y. Chew, F. Yu, A microtubule polymerase is required for microtubule orientation and dendrite pruning in Drosophila. *EMBO J.* **39**, e103549 (2020).

117. M. A. Hallen, J. Ho, C. D. Yankel, S. A. Endow, Fluorescence recovery kinetic analysis of gamma-tubulin binding to the mitotic spindle. *Biophys. J.* **95**, 3048–3058 (2008).

118. X. Li, H. Kuromi, L. Briggs, D. B. Green, J. J. Rocha, S. T. Sweeney, S. L. Bullock, Bicaudal-D binds clathrin heavy chain to promote its transport and augments synaptic vesicle recycling. *EMBO J.* **29**, 992–1006 (2010).

119. M. Buszczak, S. Paterno, D. Lighthouse, J. Bachman, J. Planck, S. Owen, A. D. Skora, T. G. Nystul, B. Ohlstein, A. Allen, J. E. Wilhelm, T. D. Murphy, R. W. Levis, E. Matunis, N. Srivali, R. A. Hoskins, A. C. Spradling, The carnegie protein trap library: A versatile tool for drosophila developmental studies. *Genetics* **175**, 1505–1531 (2007).

120. N. Karpova, Y. Bobinnec, S. Fouix, P. Huitorel, A. Debec, Jupiter, a new Drosophila protein associated with microtubules. *Cell Motil.* **63**, 301–312 (2006).

121. R. Basto, K. Brunk, T. Vinadogrova, N. Peel, A. Franz, A. Khodjakov, J. W. Raff, Centrosome amplification can initiate tumorigenesis in flies. *Cell* **133**, 1032–1042 (2008).

122. M. A. Cottee, N. Muschalik, S. Johnson, J. Leveson, J. W. Raff, S. M. Lea, The homo-oligomerisation of both Sas-6 and Ana2 is required for efficient centriole assembly in flies. *eLife* **4**, e07236 (2015).
